# Mechanistic Insights of Support Dynamics During Reactive Metal‐Support Interaction

**DOI:** 10.1002/smtd.202501950

**Published:** 2026-02-10

**Authors:** Ansgar Meise, Hiroaki Matsumoto, Marc Botifoll, Rafal E. Dunin‐Borkowski, Marc Armbrüster, Marc Heggen

**Affiliations:** ^1^ Ernst Ruska‐Centre for Microscopy and Spectroscopy with Electrons Forschungszentrum Jülich GmbH Jülich Germany; ^2^ Core Technology & Solution Business Group Hitachi High‐Tech Corporation Ibaraki Japan; ^3^ Inorganic Solid State and Material Chemistry Eduard Zintl Institute Technical University of Darmstadt Darmstadt Germany

**Keywords:** in situ, reactive metal support interaction, transmission electron microscopy, zinc palladium

## Abstract

Reactive metal‐support interactions (RMSI) are pivotal preparatory steps in the synthesis of many supported bimetallic nanoparticles. However, there is still a knowledge gap regarding the precise mechanics of such interactions, especially regarding support activation. The present study investigates the reduction of PdO nanoparticles supported on ZnO and the formation of the intermetallic compound ZnPd, using environmental scanning transmission electron microscopy (E‐STEM). The ZnO support is activated due to hydrogen spillover from the nanoparticle, which then migrates to the nanoparticle and thereby forms an encapsulation layer. Subsequently, Zn is reduced to the metallic state and diffuses into the Pd lattice at the interface, forming ZnPd and resulting in partial core‐shell structures. Structural analysis reveals that intermetallic ZnPd phase growth is diffusion‐controlled and facilitated by interfacial nucleation. These findings provide mechanistic insights into reactive metal‐support interactions, demonstrating the structural evolution of the supported catalyst system during reduction.

## Graphical abstract

This study uses in situ environmental scanning transmission electron microscopy to investigate the reduction of PdO nanoparticles on ZnO and the formation of intermetallic ZnPd. Hydrogen spillover activates the ZnO support, inducing encapsulation and Zn reduction. Subsequent Zn diffusion into Pd forms ZnPd via interfacial, diffusion‐controlled growth, elucidating reactive metal–support interactions.

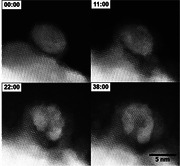

## 1 Introduction

Many metallic nanoparticles employed in catalytic reactions are typically supported on metal oxides such as Al_2_O_3_ [1], TiO_2_ [2], ZnO [3], or CeO_2_ [4]. Oxide supports are classically applied to enhance the utilization efficiency and longevity of catalysts by slowing down deactivation mechanisms and thus maintaining their fine distribution and size. However, supports should no longer be considered as inert and decoupled from the active particles. Under appropriate redox conditions, particles start to interact with their support. Such interactions between oxides and metals offer electronic, geometric, and compositional effects, which have been shown to fundamentally alter the properties of a catalytic system [5]. This phenomenon is referred to as metal‐support interaction and has attracted significant interest recently, as it can be used to tune catalytically active sites.

A chemical reaction at the oxide‐metal interface, which is activated by reducing conditions and only reversible under extremely oxidizing conditions, is known as reactive metal‐support interaction (RMSI) [6]. As RMSI frequently results in alloys and intermetallic compounds, it is a fundamental reaction in the preparation of many supported intermetallic nanoparticles [7, 8, 9, 10, 11, 12, 13, 14, 15, 16]. The large majority of the reported nanoparticle systems are based upon noble metals such as Pt, Pd, and Rh; less studied are Ni, Cu, and Ru. The preparation pathway of intermetallic compounds by RMSI is affected by their thermodynamic stability and diffusion kinetics and is therefore dependent on temperature, time, and particle size [17]. As a consequence, the resulting system often consists of a mixture of different intermetallic compounds. Such multiphase formation is facilitated by, for example, larger particle sizes [7] and formation during catalytic reactions [8, 9, 10], and occurs, for instance, in the systems Rh_3_Ti/RhTi [11], Pd_3_Si/Pd_5_Si [12, 13], and Ga_5_Pd/Ga_2_Pd_5_ [14]. Preparation protocols stabilizing a single‐phase have been established for ZnPd [15], AlPt_3_ [16], and Rh_2_Si [18], amongst others. Therefore, a mechanistic understanding of RMSI plays a pivotal role in the tunable preparation of catalytically active intermetallic compounds.

Regardless of the material system, it is generally considered that the formation of an intermetallic compound or alloy by RMSI consists of three formation steps [17]. First, hydrogen is chemisorbed on the supported nanoparticle and spilled onto the metal oxide support, which is (partially) reduced in the vicinity of the nanoparticle. Second, the reduced metal species migrates into the nanoparticle. Thirdly, the reduced metallic species are incorporated into the noble metal lattice, and the respective intermetallic compound with its characteristic structure is formed. An intermetallic system, which is prepared employing RMSI, is ZnPd supported on ZnO. ZnPd is obtained by exposing ZnO‐supported PdO particles to a reducing hydrogen atmosphere [19], a carbon monoxide atmosphere [20], or high vacuum [15]. Due to its significance as a prominent catalyst in chemical reactions, the reduction of PdO and the formation of intermetallic ZnPd are well studied, thereby an ideal example system to investigate the mechanism of the RMSI [21, 22, 23, 24, 25, 26]. Martin et al. found that PdO already adsorbed and dissociated hydrogen at a low temperature of 110 K, but was only completely reduced at room temperature [27]. As‐reduced Pd forms palladium hydride, and hydrogen is spilled onto the ZnO support. Iwasa et al. identified this hydrogen spillover by temperature‐programmed reduction [23]. They showed excessive hydrogen consumption during the reduction of PdO and remaining hydrogen on the particle‐support tandem after decomposition of palladium hydride during heating, which indicates a spillover onto the ZnO. In addition, on systems such as Pd/SiO_2_ and Pd/Al_2_O_3_, where no spillover is expected, hydrogen was completely desorbed. Activated hydrogen (partially) reduces ZnO and forms water, which is desorbed from the surface. Metallic Zn diffuses onto the nanoparticle and is subsequently incorporated in the palladium lattice, resulting in the formation of intermetallic ZnPd nanoparticles. X‐ray diffraction (XRD) and X‐ray Photoelectron Spectroscopy (XPS) measurements by Iwasa et al. provide evidence for different palladium states [23]. As‐formed ZnPd, in particular together with the ZnO support, has excellent catalytic properties in numerous reactions such as methanol steam reforming [23], formic acid decomposition [25], methanol synthesis [28], and selective hydrogenation reactions [29, 30, 31]. Besides the experimental effort, there is a lack of direct experimental evidence for the formation mechanism for supported ZnPd nanoparticles, which elucidates the local structural evolution between particle and support during reactive conditions. This is because such evidence requires a characterization method with high spatial resolution in a reactive gas atmosphere. In situ transmission electron microscopy combines both prerequisites and has been employed to characterize RMSI in a few studies.

Niu et al. studied the patterning of palladium on Pd_2_Ga surfaces employing temperature‐promoted RMSI, revealing a transition from consecutive Pd‐trimers to isolated Pd‐atoms on the surface [32]. Using in situ TEM and IR spectroscopy on adsorbed CO, they demonstrated that elevated temperatures promoted refaceting from (013)/(020) to (011)/(002) facets, driven by the incorporation of additional Ga atoms from the support. In a different study, Niu et al. investigated the formation of ZnO‐supported ZnPd nanoparticles in situ and paid special attention on the role of the intermediate phase palladium hydride [33]. The authors show that Pd transforms to β‐PdH at 50°C and to α‐PdH with increasing temperature. A complete transformation into ZnPd is reported for a 20 wt.% Pd‐loading at 340°C. In addition, they demonstrated that the intermetallic compound ZnPd favors growth along PdH*_x_* 〈111〉 due to preferential Zn diffusion. These studies illustrate the RMSI‐activated structural evolution of the nanoparticles in great detail. A combination of high‐resolution in situ TEM and illustrative models allows for a comprehension of the particle changes.

Recently, an in situ high‐resolution study of the oxidation dynamics in supported nanoparticles using aberration‐corrected environmental scanning transmission electron microscopy was provided by Chen and coworkers [34]. They demonstrated, for example, for Pd/CeO_2_, that the interfacial epitaxial match between the metal nanoparticle and support plays a dominant role in determining the oxidation dynamics. Furthermore, using identical location and in situ‐microscopy, activated CuPd/CeO_2_ supports were found to play a critical role in determining the phase separation behaviors and highlight the importance of both the phase separation of alloy nanocatalysts and in situ characterizations of catalysts under realistic gas conditions [35].

Inspired by the work of Niu et al., which elucidates the temperature dependence of the particle evolution during RMSI illustratively, this study aims to unravel RMSI‐triggered time‐resolved support activation. The utilization of time‐resolved atomic‐resolution investigation of the oxide support and particle interface at distinct temperatures is expected to facilitate a comprehensive understanding of the initial oxide‐particle interaction and the role of the support. This approach is anticipated to address the existing knowledge‐gap concerning the initialization mechanism of RMSI. This study focuses on the activation of the ZnO support and the transformation of PdO to ZnPd, with the experiments conducted in a hydrogen atmosphere in situ at ambient temperature and at 200°C. Furthermore, the effect of different reaction parameters such as the electron beam, temperature, and hydrogen pressure on the material system is discussed.

## 2 Results and Discussion

**FIGURE 1:**
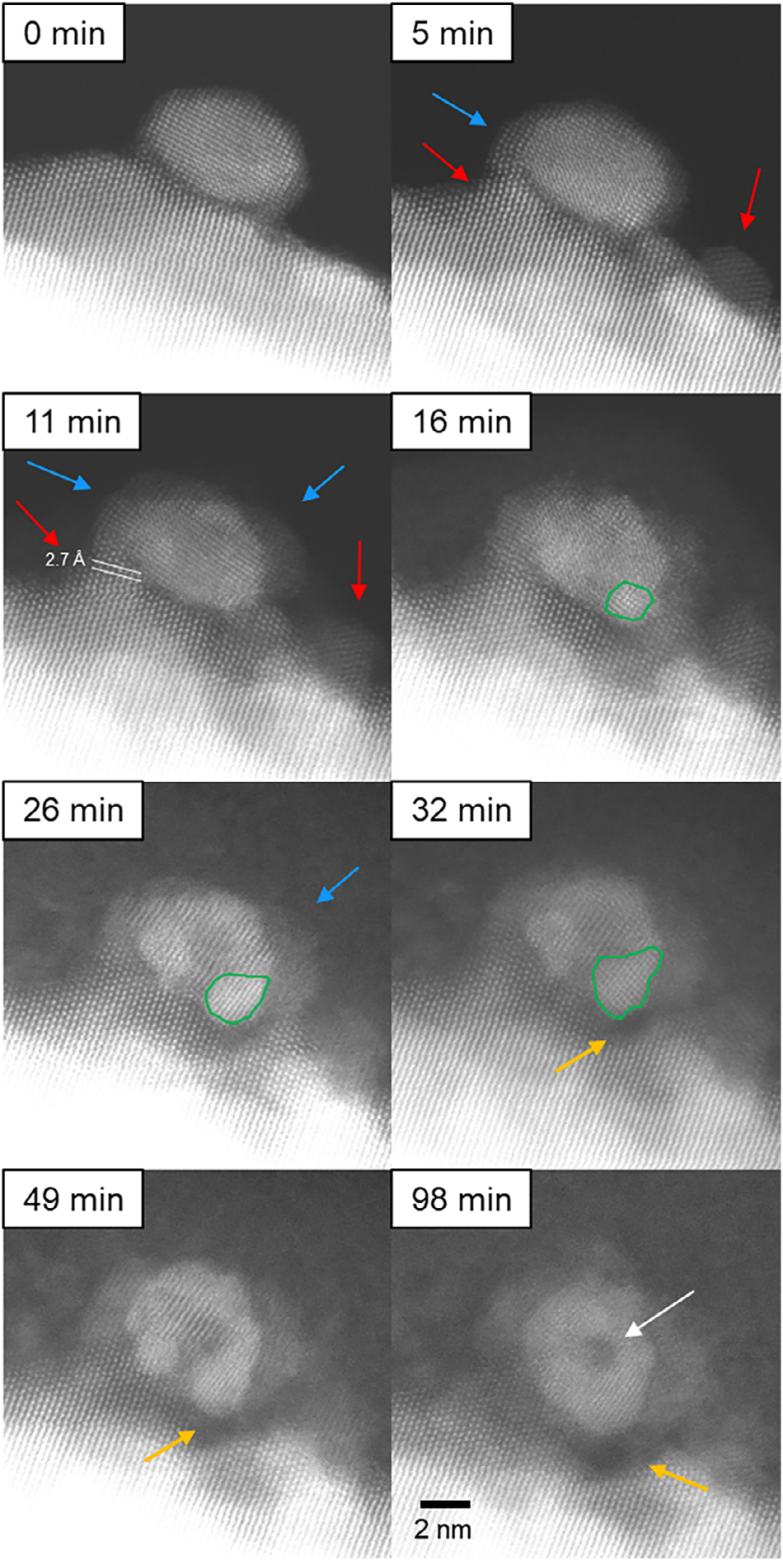
Dark field STEM image series of the structure transformation of the nanoparticle‐support system during reduction in a hydrogen atmosphere at room temperature. Structural changes are highlighted by arrows. Red arrows indicate the formation of ZnO enrichments on the support surface. Blue arrows highlight the encapsulation of the nanoparticle by ZnO. The green outlines illustrate the formation and growth of a new phase. The orange arrows indicate ZnO reduction and Zn incorporation into the nanoparticle. The white arrow highlights a remaining nanoparticle core showing incomplete particle transformation.

**FIGURE 2:**
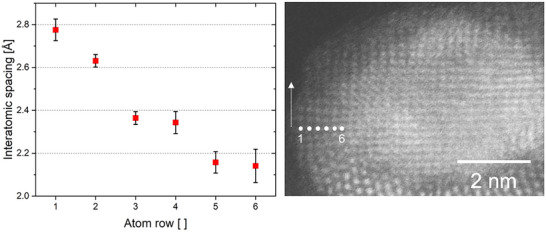
Clipped DF STEM image of supported PdO after 11 min of hydrogen exposure. The original image is displayed in Figure 1. Interatomic spacings are measured along the row of atoms in the arrow's direction. Atom rows are numbered from left to right as displayed. Interatomic spacings of 4 to 5 atoms are measured three times and are averaged. The error is given by the standard deviation.

PdO nanoparticles supported on ZnO (5 wt.% Pd loading) were prepared following the synthesis described by Iwasa and coworkers [36]. As‐prepared samples were reduced in situ in a hydrogen atmosphere corresponding to a specimen pressure of ∼1.5 Pa at ambient temperature. Two distinct sample locations are monitored during the reduction experiment. Figures 1 and 4 show a dark‐field scanning transmission electron microscopy (DF‐STEM) image series of structural changes of the nanoparticle‐support system during an in situ experiment conducted in a hydrogen atmosphere at ambient temperature. Figure S1 shows a DF‐STEM image of the pristine nanoparticle‐support structure. An artificial intelligence‐assisted phase identification is conducted, which identifies the pristine nanoparticle as PdO phase aligned along [1 1 0] and the support as hexagonal ZnO aligned along [1 1 0] [37]. Furthermore, XRD (Figure S2) measurements confirm the presence of PdO and ZnO. Exposure to the electron beam introduces no significant structural changes to the particle‐support system during imaging in vacuum. The corresponding bright‐field (BF) STEM images are shown in Figures S4 and S7, and secondary electron (SE) STEM images in Figure S9. The initial sizes of the particles in Figures 1 and 4 are 7.1 × 4.3 nm^2^ and 14.3×9.4 nm^2^, respectively. After 5 min, the support morphology changes considerably, and a coarse surface develops. As highlighted by the red arrows, ZnO piles up at the surface and partially envelopes the nanoparticle (blue arrows). A variation of the DF contrast levels of the ZnO support is furthermore observed as the in situ reaction progresses. The image contrast of the support in contact to the nanoparticle is similar to the initial state, indicating a migration of ZnO to the nanoparticle or the subsidence of the nanoparticle into the support. Other support regions show weaker SE and BF contrast, suggesting thinning or compositional changes. The present changes in surface morphology occur preferably in the perimeter of the nanoparticle. In Figure 1, it is clearly seen that ZnO migrates from the support and progressively covers the left side of the PdO nanoparticle. In the area close to the nanoparticle (Figure 1, 11 min) an increase of the [0 0 1] lattice spacing by 3.5% from 5.2 ± 0.1 to 5.5 ± 0.1 Å, and a [1 ‐1 0] spacing decrease by 3% from 2.8 to 2.7 Å is observed, indicating a straining of the hexagonal ZnO structure. After 11 min, an interaction between the nanoparticle and support is activated, as displayed by the blue arrows in Figure 1, in such a way that a continuous transition zone with respect to interatomic spacings and crystal structure of nanoparticle and support is found. To estimate the change in interatomic spacing, six atomic rows are labelled and measured along the direction of the arrow from the position marked, as shown in the sectioned DF STEM image in Figure 2. The interatomic spacings vary from ∼2.8 Å at the edge to ∼2.1 Å at the interface between support and nanoparticle. The interatomic spacings at the outer and inner perimeter match well with those of ZnO and PdO, respectively. Furthermore, a strain analysis of the particle and support at 0 and 11 min is performed (Figure S6). The analysis reveals the presence of a high compressive strain of 18.7% in the ZnO interface region.

**FIGURE 3:**
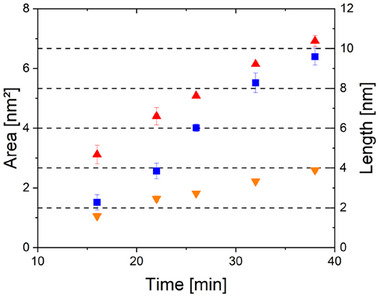
The growth of the phase is quantified by measurements of area (blue squares), circumference (red triangle), and Feret's diameter (orange triangle). The area is given in nm^2^ on the left axis. The length is given in nm on the right axis. An example of the phase measurement is represented in Figure S10.

After 16 min, the PdO nanoparticle is largely encapsulated by ZnO. Once formed, the coverage layer has a constant thickness that hardly changes over time. For instance, the thickness of the coverage layer is ∼1.48 ± 0.1 nm after 16 min and ∼1.58 ± 0.1 nm after 32 min. The lattice spacing of the coverage layer on the nanoparticle corresponds to that of ZnO. Intriguingly, the support close to the nanoparticle vanishes after 22 min, as indicated by the orange arrows. While in the first minutes of reduction, the support is mainly affected by the reducing atmosphere, subsequently, structural changes in the PdO nanoparticle start to evolve. Nucleation of a new phase, represented by an area of brighter contrast in the DF STEM images, occurs at the interface between nanoparticle and support, as highlighted in green in Figure 1. A phase analysis using a fast Fourier transform (FFT) of the respective area shows the presence of the metallic Pd phase at 16 min (Figure S5). To estimate the growth of the Pd phase area, its perimeter is manually determined three times per image, and average values for area, circumference, and Feret's diameter are determined with the respective standard deviations as errors. For a better understanding of the measurement procedure applied, examples of shapes are marked in Figure S10, and the corresponding values are displayed in Figure 3. A continuous growth by ∼0.1 nm/min continuously over time is found.

The interatomic spacings within the green areas between 16 and 32 min are presented in Figure S11. In accordance with the FFT analysis, the lattice spacings initially match best with that of elemental Pd, but increase with time, in particular along the b direction (Figure S11). An FFT phase analysis of the area at 32 min in presented in Figure S5. The identification of a phase becomes somewhat ambiguous. A best match is found with the Pd_2_Zn Pnma phase (621) zone axis. The observed angle of 79° between planes, however, refers to a heavily strained cubic or a tetragonal/orthorhombic phase. It is hypothesized that Pd incorporates Zn, progressively deforming the cubic unit cell. In accordance with our strain analysis presented in Figure S6, we hypothesize that the reduction and transformation toward the intermetallic ZnPd phase may occur via highly strained continuous alloying and not via the formation of intermediate intermetallic equilibrium phases.

Interestingly, the growth of the phase marked by green outlines is accompanied by the dissolution of adjacent ZnO, described above. At the left‐hand side of the nanoparticle, a second bright evolving feature indicates the growth of another phase. The orange arrows indicate ongoing ZnO reduction. Growth of individually formed phases leads to their assembly and formation of a core/inner‐shell/outer‐shell structure, as highlighted by the green arrows at 57 min. The centre of the nanoparticle still shows a dark contrast crystal structure, as indicated by a white arrow. and is coated with presumably‐formed ZnPd resulting from the reaction of Pd with elemental zinc formed by the reduction of ZnO. Comparison between the state at 64 and 98 min indicates that prolonged exposure to a reducing atmosphere does not trigger any further significant structural changes.

**FIGURE 4:**
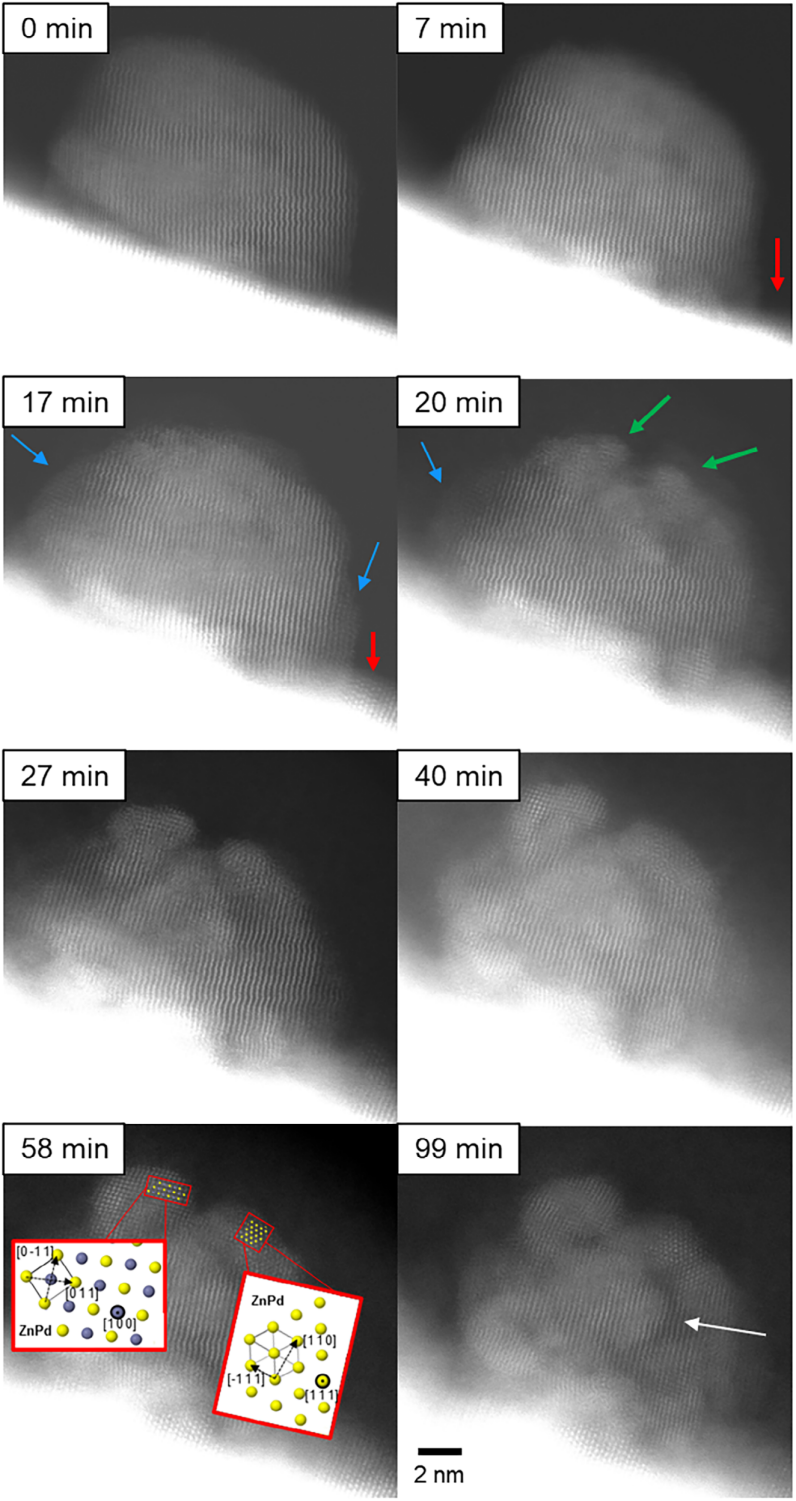
Dark field STEM image series of the structure transformation of another nanoparticle‐support system during reduction in a hydrogen atmosphere at room temperature. Structural changes are highlighted by arrows. Red arrows indicate the formation of ZnO enrichments on the support surface. Blue arrows highlight the encapsulation of the nanoparticle by ZnO. The green arrows illustrate the formation and growth of a new phase. The white arrow highlights incomplete particle transformation. Lattice spacings measured are in good agreement with the ZnPd crystal structure displayed from reference [36].

Figure 4 shows a DF image series at a different location of the specimen during the identical in situ reduction experiment as shown in Figure 1. Initially, the nanoparticle shows a single crystalline PdO structure, and the ZnO support is mostly flat. Similar to the results described above, the ZnO support evolves over time during reduction. As indicated by red arrows, a coarsening of the ZnO support is observed, and ZnO clusters are formed on the support surface. The ZnO cluster grows along [1 ‐1 0] and partially envelopes the nanoparticle (blue arrows). After 17 min, the nanoparticle surface is partially decorated by crystalline ZnO islands. Similar to the results discussed above, nucleation and growth of new phases are observed here as well. After 20 min, the single crystalline structure of the observed nanoparticle is distorted by the emergence of crystal nuclei, which are visible at the locations indicated by green arrows in Figure 4. In general, nuclei of new phases are formed in close proximity to ZnO surface layers or islands. The novel phases continue to grow over time and slowly transform most of the nanoparticles. After 58 min, the novel phases are the dominant feature on the top of the nanoparticle. The crystal structure on the left agrees well with the ZnPd phase aligned along [1 0 0], as demonstrated by the model in Figure 4. The measured lattice spacings are 2.1 ± 0.1 and 2.2 ± 0.1 Å, corresponding to ZnPd (0 1 1) of 2.18 Å. At the right‐hand side, a ZnPd phase region aligned along [1 1 1] is identified. Here, the lattice spacings of 4.0 ± 0.1 and 2.7 ± 0.1 Å are measured and match with ZnPd (1 1 1) of 2.64 Å and (1 1 0) of 4.09 Å. Other parts of the nanoparticle still show the pristine crystal structure, as marked by the white arrows, indicating only a partial transformation.

We note that although we tried to minimise the electron beam exposure during the experiment by closing the gun valve when no images were being recorded, we cannot rule out a partial reduction of the sample by the electron beam. In order to evaluate the electron beam effect on the PdO nanoparticles, we have exposed PdO particles with the same electron dose as in the reduction experiments (Figures 1 and 4) in a series of 12 consecutive image acquisitions at ambient temperature in vacuum. This experiment shows evidence of a slight structural transformation due to electron beam irradiation (Figures S14 and S15), demonstrating that the influence of the electron beam during the reduction experiments cannot be neglected. In order to further decouple the electron‐beam‐induced effect from the reduction in gas environment, we have analyzed images of reference locations (Figure S16), which were only imaged before and after the exposure to hydrogen but not during the in situ experiments. In this way, the exposure to the electron beam was minimized to only two images. The analysis of these reference locations shows the local presence of PdO before and ZnPd after the reduction.

Three major differences between the present in situ E‐STEM experiment and conventional reduction experiments under ambient laboratory conditions have to be considered: The presence of an electron beam, a lower reduction potential due to low partial pressure and a lower temperature (see Supporting Information, section entitled *Effects induced by the Experimental Conditions*). We note that the total H_2_ specimen pressure in our experiment is 1.5 Pa, which is about 5 orders of magnitude lower compared to typical pressures during catalytic reactions. Electron beam effects may affect the present in situ reduction experiment by introducing additional lattice defects and increasing the temperature of the sample. However, activation and transformation of reference regions, which were only imaged before and after reduction, indicate that the mechanisms derived may be facilitated but not triggered by the electron beam. In addition, similar results between *ex* and in situ experiments may indicate only a small effect of the pressure difference in the present case. In addition, in situ STEM reduction experiments of supported PdO were conducted at 200°C (see Supporting Information, section entitled *Reduction of Palladium Oxide at 200°C*). Under such conditions, only the kinetics are accelerated, while the mechanism stays the same, and the nanoparticles are encapsulated only within a few minutes. The conditions under which nanoparticles are encapsulated by SMSI, as described in the literature, are extremely diverse. Possible influencing factors are, among others, the materials system, the particle size, gas pressure, and temperature. For an overview of the factors that can influence encapsulation, please refer to recent review articles on the subject [38, 39, 40, 41].

Our experimental results reveal a complex evolution of the support‐nanoparticle system during in situ reduction. Despite the occurrence of numerous simultaneous structural changes, the reaction can be divided into two distinct steps: the activation of the ZnO support and the transformation of the nanoparticles. We will discuss the two subsequent steps in the following.

### 2.1 Activation of the ZnO Support

Our phase identification based on STEM imaging and XRD measurements confirms that the pristine system consists of PdO nanoparticles supported on ZnO. The absence of XRD reflections representing palladium species can be explained by the small crystallite size and low loading. Potential PdO reflections are weak and broad, thus making them hardly distinguishable, as shown at ∼34°. Thus, the occurrence of other Pd species such as Pd, PdH*_x_* or ZnPd cannot be excluded, and minor quantities might be present.

**FIGURE 5:**
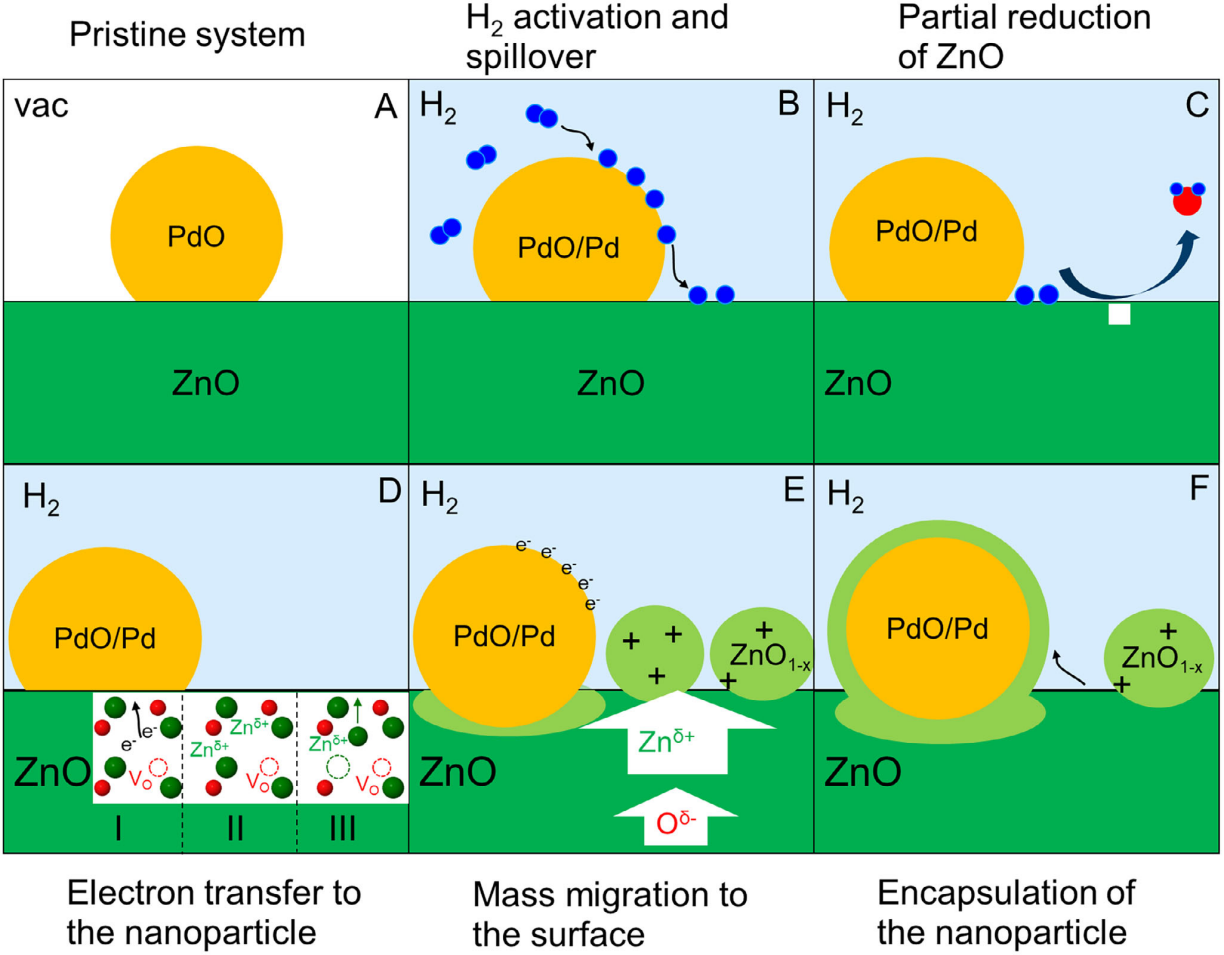
Schematic illustration of the surface activation mechanism during reduction. Zn is green, H blue, and O red. A: Pristine PdO particle supported on ZnO. B: Hydrogen is activated on PdO and spilled onto the support. C: On the ZnO surface, hydrogen reacts with the oxide ions and forms water. Water desorbs from the surface, and oxygen vacancy V_O_ is generated. D: Coordinatively unsaturated Zn species donate delocalized electrons to the system, which transfer to the nanoparticle (I). Positive Zn species form a positive net space charge in ZnO (II). The energetically unfavorable positive net space charge is minimized by Zn‐migration to the surface (III). E: Subsequently, migration of negatively charged oxygen species is activated, and oxygen‐deficient ZnO_1‐_ *_x_* clusters form on the surface. F: The negatively charged nanoparticle is encapsulated by positively charged Zn*_x_* O to minimize the surface energy of the system.

At room temperature, the ZnO support changes strongly during the initial 5 min of reduction. This is rather surprising, given that pure ZnO is known to resist reduction at such low temperatures. Thus, a direct activation of hydrogen by ZnO is unlikely and an interaction between the nanoparticle and support must be considered. In contrast to ZnO, PdO is known to adsorb and dissociate hydrogen already at a temperature of 110 K [27]. Activation of hydrogen on the surface of the PdO nanoparticles is therefore more probable than on ZnO directly. The activation mechanism is schematically illustrated in Figure 5 and is comparable to the SMSI model introduced by Fu et al. for the encapsulation of Pd by TiO_2_ [42]. Hydrogen species activated on the PdO surface migrate due to spillover from the nanoparticle to the ZnO surface. The existence of such hydrogen spillover effects from a noble metal to ZnO has long been known in methanol synthesis with Cu catalysts and is also expected here [43]. ZnO is partially reduced by spilled‐over hydrogen. Hydrogen combines with oxygen to form water, which is desorbed from the ZnO surface, and oxygen vacancies are generated. Due to the missing oxygen, zinc states around the vacancy are unsaturated and donate delocalized electrons to the system. Such an electron excess around the oxygen vacancies increases the Fermi‐level and leads to an n‐type doping of ZnO – a phenomenon described for ZnO earlier [44]. The electronic structure of semiconducting ZnO is also determined by the composition and electronic nature of the supported nanoparticle.

The work function *W* of materials depends on their Fermi‐level *E_F_* (*W*  =  *e*ϕ −  *E_F_*), where *e* is the charge of an electron and ϕ is the electrostatic potential in the vacuum. Since Pd and PdO have with 4.8‐5.6 eV and 6.04 eV, respectively, a higher work function than ZnO with 4.6 eV [45], their Fermi‐level is lower. Consequently, the electrostatic equilibrium at the interface between the support and the nanoparticle is distorted. The present interface is similar to a Schottky diode, where a metal and an n‐type semiconductor form a junction. The equilibrium at the junction is restored by an electron transfer to the nanoparticle. The nanoparticle becomes negatively charged, and the bands of ZnO are upbent, resulting in an electron depletion zone. Due to oxygen vacancies, ZnO is defect‐rich and has a positive net space charge. The electrostatic attraction by the negatively charged nanoparticle promotes outward diffusion of positively charged Zn species and inhibits the transport of negatively charged oxygen species to the surface at low temperature. The mass transport at ambient temperature is therefore activated by a combination of the present electrostatic force and the defect‐rich structure. Zn‐enrichment on the surface lowers the positive net space charge in bulk ZnO, and oxygen ions start to migrate to the surface, leading to a relocation of ZnO on the surface. Since sufficient oxygen diffusion, which is a prerequisite for stochiometric formation, is only achieved at higher temperatures, oxygen‐deficient ZnO_1‐x_ (x < 1) is formed at room temperature. Such clusters are present in Figures 1 and 4. Their lower contrast can be explained by their lower thickness. The driving force for the coverage is thus very similar to the one for the solid‐solid wetting, i.e., minimization of the surface energy [46]. Such mass transport in a single atomic or liquid‐like movement is activated by a difference in surface energy and charge between the nanoparticle and ZnO. Pd and PdO have a higher surface energy than ZnO [47, 48, 49]. In order to minimize the total surface energy of the system, it is advantageous if ZnO covers the nanoparticle. This coverage is facilitated in reducing the atmosphere only as this builds up the electrostatic driving force between the negatively charged nanoparticle and the oxygen‐deficient ZnO_1‐x_, which has a positive net charge. After ∼20 min, ZnO shows no further change, indicating an equilibrium. This is in good agreement with the model derived above. The nanoparticle is now covered by ZnO, which reduces the surface energy and neutralizes the charge. Thus, the system is in an energetically favorable state with no apparent driving force for ZnO migration. This is reinforced by the coverage layer of the nanoparticle, which stops growing after being a few atoms thick. If there were an additional driving force, a continuous growth of the layer would be anticipated. However, this is not the case.

The interface between the nanoparticle and covering layer is presumably due to Pd‐Zn or Pd‐O‐Zn interactions. Given that the undistorted crystal structures of the interfacial regions are Pd and ZnO, the transition phase reduces the stress induced by the difference in lattice parameters. These interfacial interactions might act as a nucleus for the formation and growth of intermetallic compounds in RMSI, especially if they are of the Pd‐Zn type. The existence of such bimetallic interfaces was already proposed by Tauster et al., who assigned the SMSI‐related loss in chemisorption to the formation of metal‐metal bonds at the interface during reduction [50].

At 200°C, the activation of the support is accelerated. As the encapsulation of the nanoparticles is completed within the first 3 min of reduction, the temporal resolution of image acquisition was too poor to dissect the individual steps of the activation mechanism. Due to rapid and complete encapsulation, it can be concluded that surface diffusion is significantly enhanced by an increase in temperature. Simultaneously, the temperature effect on bulk diffusion is [51, 52] as the rate of ZnO cluster formation at the surface is similar to that of room temperature, which indicates a similar diffusion rate for outward migration of positive Zn species. In addition, encapsulation may have a detrimental effect on outward diffusion as it covers the negatively charged nanoparticle and thus reduces the electrostatic attraction for positive Zn species. The correspondence between the thickness of the coverage layers supports the assumption derived above that minimization of surface energy is the driving force for encapsulation. In general, the activation mechanisms of the support at ambient temperature and 200°C are in good agreement. The different kinetics of the reactions can be well explained by the temperature difference.

Although the pristine structure of the nanoparticle corresponds to PdO, the nature of the nanoparticle, i. e. if it is PdO or Pd, remains uncertain until ZnPd forms. The in situ images acquired do not allow a clear assignment of the structure to a specific Pd compound. However, the increase in Z‐contrast during reduction indicates a compositional change, suggesting a gradual reduction of PdO to elemental Pd. The occurrence of a transformation is supported by the fact that PdO can be reduced at ambient temperature [27]. In addition, the PdO nanoparticles were observed to undergo rapid reduction at 200°C. Consequently, the formation of elemental Pd is probably an outcome of the reduction process, despite the absence of definitive structural evidence.

Palladium hydride, PdHx, exhibits two phases depending on the atomic ratio x. The region 0< x ≤ 0.03 corresponds to the α‐phase, while in the region of x ≥ 0.58 the β‐phase is formed. The coexistence of both phases is observed in the region in between. The β‐phase possesses the same symmetry as the initial (α‐)Palladium fcc lattice, but the formation of the cubic β‐phase (*a* = 0.4035 nm) during hydrogen uptake of the lattice leads to an increase of the cell parameter compared with the α‐phase Pd (0.3891 nm) [53, 54]. A wide range of factors can influence palladium hydride formation during the reduction of PdO in a hydrogen atmosphere. Factors promoting hydride formation include larger metal surface area, higher surface roughness/defect concentration, hydrogen pressure, as well as temperature. On the contrary, hydride formation can be inhibited by several mechanisms: alloying with rhenium or gold suppresses β‐PdH*_x_* formation, surface overlayers, and maintaining hydrogen pressures below the α‐to‐β phase transition [55, 56, 57, 58, 59, 60]. For example, Tew and co‐workers [57] reported a size‐dependency on the formation of PdH*_x_*. Smaller nanoparticles provide fewer interstitial places for the hydride formation and therefore incorporate less hydrogen in their lattice. In contrast, due to their higher surface‐to‐bulk ratio, smaller Pd nanoparticles have a higher relative surface adsorption.

Bugaev et al. [54] have investigated the process of hydride formation of Pd nanoparticles in a wide pressure range using volumetric measurements with simultaneous EXAFS and XRPD experiments. The results demonstrate that, in the pressure regime below 1000 Pa, Pd nanoparticles remain in their α‐phase structure. The authors explain their result by considering the α‐phase to contain a very low amount of hydrogen, which does not contribute significantly to the structural disorder. At higher pressures of about 2000 Pa, a phase transition to the β‐PdH*_x_* phase and an expansion of the lattice set in.

Niu et al. [32] investigated the formation of ZnO‐supported ZnPd nanoparticles using in situ transmission electron microscopy and identified the formation of the β‐PdH*_x_* intermediate phase. The β‐phase was identified by measuring a slight lattice expansion using FFT and direct lattice plane measurement of HRTEM images. Most importantly, the experiments were performed using a closed‐cell gas heating holder setup (DENSsolutions Climate) operated at 70 kPa sample pressure using a 10 vol.% H_2_ /He gas mixture. The resulting H_2_ partial pressure is thus significantly higher than the pressure in our current experiments and, as reported by Bugaev, above the level of transformation from the α‐ to the β ‐phase. In our current experiments, we used an open‐cell environmental STEM configuration with a comparably low H_2_ partial pressure of 1.5 Pa. This pressure level is considerably below the α‐/β ‐phase transformation level, which is consistent with the presence of the α‐Pd or α‐PdHx phase.

### 2.2 Transformation of the Nanoparticles

While the ZnO support was subject to strong structural evolution during the first part of the reaction (< 20 min) at ambient temperature, the individual nanoparticles were largely unaffected by the reactive atmosphere. However, after the encapsulation of the nanoparticle, new phases nucleate at the Zn‐Pd interface. Intriguingly, all nuclei are formed at the interface, indicating that the interface favors heterogeneous nucleation. The intermetallic compound ZnPd has a wide compositional existence range in the Pd‐Zn phase diagram, and its lattice parameters exhibit only a small mismatch to cubic Pd [15]. Zn‐Pd heteronuclear bonds are stronger than homonuclear Zn‐Zn and Pd‐Pd bonds, while the latter are the driving force for the tetragonal distortion [61, 62]. Thereby, substitution of 50% of the Pd atoms in the Pd (111) surface by Zn leads to tetragonal ZnPd. As ZnPd formation is thermodynamically favored [63], its formation might be kinetically hampered due to slow diffusion at ambient temperature.

**FIGURE 6:**
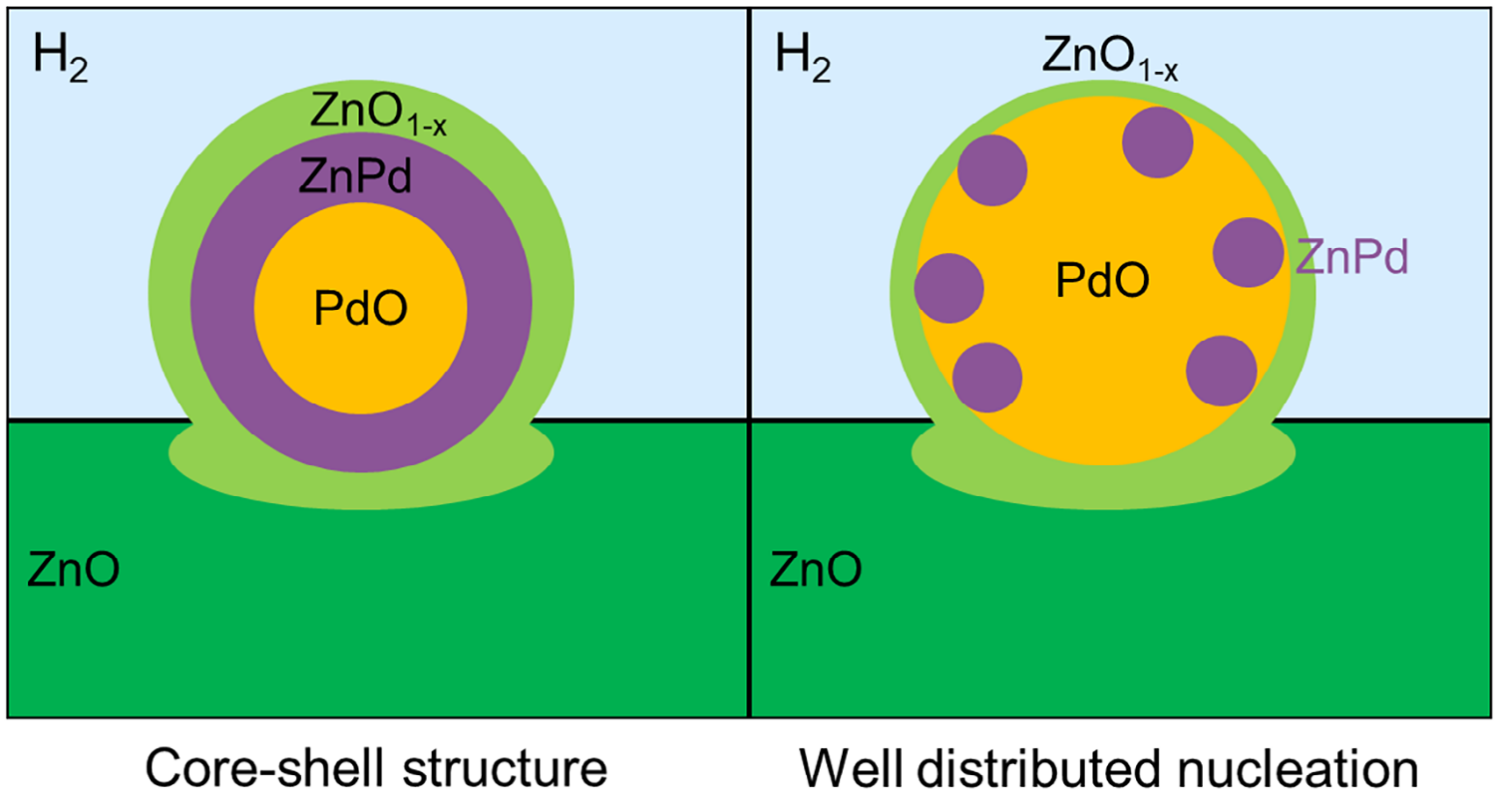
Schematic sketch of the different transformation types observed of the supported PdO nanoparticles: a core‐shell structure and a well‐distributed nucleation. The differences in nucleation are based on kinetic limitations due to the low diffusion at ambient temperature and compositional changes.

The observed phase grows with an average rate of 3.7  · 10^−3^ *nm* ^2^/*s* and 3.7  · 10^−17^ *cm* ^2^/*s* respectively between 16 and 38 min, as illustrated in Figure 3. Since there is no literature data available for the diffusion of Zn in Pd or ZnPd, we use Cu and CuZn as a reference for our estimation, given its similarly large unit cell (Cu a = 3.62 Å [64]; Pd a = 3.89 Å [65]). Kuper et al. reported a diffusion coefficient of 2.87  · 10^−13^ *cm* ^2^/*s* for Zn in copper‐zinc at 264°C [66]. Eastman et al. found similar values for interdiffusion coefficients for the same system in the range of 10^−12^ *cm* ^2^/*s* at 100°C [67]. Assuming that the given Arrhenius plots can be extrapolated to ambient temperature, the growth rate has about the same order of magnitude as the anticipated diffusion coefficient at room temperature. Since the diffusion coefficients depend on composition and temperature, the approach should be considered a guideline only that semi‐quantitatively supports the assumption that growth is controlled by diffusion. Incorporation of Zn into the Pd lattice is also supported by the reduction of ZnO at the interface. Continuous loss of ZnO image contrast indicates a decrease in thickness. Such behavior is exclusively found in the vicinity of a growing phase. After ∼ 60 min, the multicrystalline structure features of the novel phases correspond well to ZnPd. Small deviations in size and angle from the structure model can be explained by the accuracy of measurement, the broad compositional existence range of ZnPd, the small particle size leading to a high surface to volume ratio, the strain induced by contact of different crystal structures, and potential imaging artefacts such as de‐scan, calibration inaccuracy, and residual lens aberrations. Intriguingly, residual parts of the nanoparticle still have the pristine crystal structure and image contrast corresponding to the PdO phase. Such pristine PdO phase regions are predominantly found in the centre of the nanoparticle, while the ‘skin’ is transformed to ZnPd, which leads to a core‐shell structure. The observed nanoparticles transform differently (core‐shell structure vs. well distributed nucleation), indicating the existence of a size effect, as illustrated in Figure 6. The present formation can be explained by kinetic limitations at ambient temperature and compositional changes. Given that most Zn is provided from the interface directly, Zn penetrates into Pd, diffuses through the Pd lattice, and forms ZnPd. As the diffusion coefficient of Zn in ZnPd is supposedly smaller than in Pd (caused by the missing thermodynamic driving force), assuming a similar diffusion coefficient for Zn as in CuZn, the already small diffusion at ambient temperature is further reduced and now hampers any substantial Zn transport to the centre.

Interestingly, the nucleation transformation is only observed in the nanoparticle, but not in the encapsulating ZnO layer. Assuming that Cu in ZnO has similar diffusion properties to Pd, its diffusion rate in ZnO is significantly lower than that of Zn in the Pd lattice (10^−12^ *cm* ^2^ *s* ^−1^ compared to 10^−6^ *cm* ^2^ *s* ^−1^ at 1000°C) [66, 68] as the diffusion in Pd is thermodynamically driven by the rather strong interaction of the two metals [61], suggesting that inward diffusion of Zn species is dominant over outward diffusion by Pd species into ZnO. Such behavior, in which the diffusion rate of a semiconducting species at an interface is faster than that of its metallic counterpart, is known as the reversed ‘Kirkendall’ effect [69] and also applies here.

First evidence for the alloy formation of Pd/ZnO catalyst was given by Hong et al. [19]. and Zsoldos et al. [70]. using XRD, TPR, and XPS. The large positive shift found in the XPS measurements points out that the electronic state of metallic Pd is strongly influenced by Zn in the alloy, which probably results in an electron‐deficient state of Pd. Furthermore, reduction experiments on Pd/ZnO were carried out over with different Pd loadings [71, 72]. Results using XRD and XPS showed that the intermetallic ZnPd phase was selectively produced during Pd/ZnO reduction. It was suggested that hydrogen retained in Pd spilled over to ZnO. The ZnO was reduced to metallic Zn, being converted to ZnPd. Iwasa et al. also showed that the loaded Pd was completely transformed to the ZnPd alloys upon reduction. The in situ reduction of Pd on Titania was furthermore reported by combining in situ transmission electron microscopy and density functional theory calculations [73]. The authors demonstrate that an amorphous reduced titania layer is formed at low temperatures, and that crystallization of the layer into either mono‐ or bilayer structures is dictated by the reaction environment and predicted by theory. An amorphous TiOx layer is initially formed at low temperature (∼300°C) in a reducing atmosphere, followed by crystallization into an ordered layer epitaxial with the Pd(111) surface at ∼500°C. With reference to well‐known properties of the SMSI, the amorphous layer corresponds to the state produced by low‐temperature reduction, whereas the crystalline layer corresponds to the state produced by high‐temperature reduction.

At 200°C, rapid nanoparticle structure transformation from PdO to Pd is observed, revealing a strong kinetic influence. Reduction of PdO in certain morphologies, e.g., small particles, can already be activated by heating in vacuum alone, as elemental Pd is found in some nanoparticles before hydrogen is injected. Such behavior emphasises the energetically favorable reduction of PdO at 200°C. Accordingly, it is likely that at the sample location studied in situ at 200°C, single‐crystalline Pd is formed even before hydrogen is first injected. While the right‐hand side of the nanoparticle remains unchanged during reduction, the left‐hand side transforms to ZnPd (Figures S17–S19). Dissolution of neighboring ZnO may be an indicator for Zn incorporation into the Pd lattice. Reference regions support the model of partial reduction, as the darker core in DF indicates a lower elemental mass and heterogeneous element distribution in the nanoparticle (Figure S20). Such partial transformation, which was also found during reduction at ambient temperature, is in correspondence with the work by Penner et al. [15]., studying the reduction of supported Pd nanoparticles ex situ at 1 bar hydrogen pressure. They demonstrated that Pd nanoparticles at a temperature of 250°C predominantly occurred in a partially alloyed state, forming a ZnPd skin but retaining the elemental Pd core. At a temperature above 323°C, a complete transformation of Pd into ZnPd was achieved. Formation of ZnPd could also be found at the lowest tested temperature of ∼200°C, which is consistent with the work by Iwasa et al. [14, 74]. The correspondence between the present in situ work and the absence of ZnPd reflections in the XRD and the corresponding XPS data obtained by Iwasa et al. [74] after reduction at ambient temperature can be explained by closely neighboring reflections and the thinness of ZnPd of 1 – 2 nm, which broadens the XRD reflections. In addition, the ZnPd XPS signal might be superimposed by observed Pd and PdO, and by a higher loading of the sample, favoring larger particles and thus the core species.

## 3 Conclusion

The reduction of PdO nanoparticles supported on ZnO and the formation of the intermetallic compound ZnPd was studied using E‐STEM and can be divided into two regimes. The first regime comprises the activation of the ZnO support by hydrogen spillover, ZnO migration to the surface, and an encapsulation of the nanoparticle. The second regime includes the nucleation of the ZnPd phase, its diffusion‐controlled growth, and the formation of a core‐shell structure due to kinetic limitations that are in good agreement with earlier *ex situ* experiments. As a result, ZnPd nanoparticles with a thin ZnO shell are formed, representing an ideal precursor state for the ZnPd‐ZnO teamwork necessary i.e. in methanol steam reforming. The electron beam may enhance the effects of the reduction but not trigger them as shown by reference regions and the stability under pure electron beam exposure. Other potential effects induced by the experimental conditions, such as the formation of hydrogen plasma and temperature differences, have a minor impact and can largely be neglected. The in situ results represent the first direct observation of the reduction of PdO and the respective RMSI. The mechanism has many similarities with SMSI but is different in the interaction between the nanoparticle and coverage layer, as the metal particle is transformed into an intermetallic compound by the RMSI.

## Conflicts of Interest

The authors declare no conflicts of interest.

## Supporting information




**Supporting File**: smtd70547‐sup‐0001‐SuppMat.docx.

## Data Availability

The data that support the findings of this study are available from the corresponding author upon reasonable request.
