# Supplementary material for: Mechanistic Insights of Support Dynamics During Reactive Metal‐Support Interaction
Source: Small Methods. 2026 Feb 10;10(5):e01950. doi: 10.1002/smtd.202501950 (PMC12972270; doi:10.1002/smtd.202501950)
Supplement: Supplementary file 1 — Supporting File: smtd70547‐sup‐0001‐SuppMat.docx. [file SMTD-10-e01950-s001.docx]

SUPPORTING INFORMATION

Mechanistic Insights of Support Dynamics during Reactive Metal-Support Interaction

Ansgar Meise^1^, Hiroaki Matsumoto^2^, Marc Botifoll^1^, Rafal E. Dunin-Borkowski^1^, Marc Armbrüster^3^, Marc Heggen^1^*

^1^ Ernst Ruska-Centre for Microscopy and Spectroscopy with Electrons, Forschungszentrum Jülich GmbH, 52428 Jülich, Germany

^2^ Core Technology & Solution Business Group, Hitachi High-Tech Corporation, Ibaraki, Japan

^3^ Inorganic Solid State and Material Chemistry, Eduard Zintl Institute, Technical University of Darmstadt, 64287 Darmstadt, Germany

* Corresponding author E-mail: m.heggen@fz-juelich.de

Experimental

Samples with a targeted palladium loading of 5 wt.-% were prepared by incipient wetness impregnation following the protocol reported by Iwasa *et al.*^[1]^ This involved the dissolution of Pd(NO_3_)_2_ ⋅ x H_2_O (Sigma-Aldrich) in the appropriate amount in water. After impregnation, samples were dried at 110 °C for five days. Subsequently, the sample was calcined at 500 °C for 3 h in O_2_:N_2_ (20:80). The as-prepared palladium oxide supported on zinc oxide was transferred to a MEMS heating chip fabricated by NORCADA (Edmonton, Canada). *In-situ* reduction experiments were conducted, employing E-STEM on a Hitachi High-Technology HF5000, operating at 200 kV. Hydrogen was injected with a mass flow of 2.5 sccm resulting in a column pressure of $1.5\text{ }\cdot{10}^{\text{-}2}$ Pa, which corresponds to a specimen pressure of ~1.5 Pa. The reduction was performed at ambient temperature and 200 °C for 100 min with a freshly prepared sample of supported palladium oxide. The regions of interest were only exposed to the electron beam during image acquisition and focus alignment to reduce electron beam effects to a minimum. The imaged regions were therefore not observed in between image acquisition. In addition, some locations were only imaged before and after the reduction, to evaluate the effect of the electron beam. The electron dose per image is determined according to Egerton^[2]^ and is around ~ 10^7^ e^-^/Å².

The *in-situ* STEM images were processed in a frame by frame basis by selecting representative frames in which key steps of the transition were captured. Each frame was first segmented to separate the distinctive material regions from the background. The resulting segmented atomic resolution crops were processed with an automated artificial intelligence-assisted method to extract the most likely crystallographic phase explaining the experimental disposition of observed and time-evolving crystallographic planes. The database of crystal phases was taken as a subset of the inorganic crystal structure database (ICSD), refined with the crystal phases that physically align with the temperature and pressure conditions of the involved reactions. The complete details of the processing can be found in the following references.^[3,4]^


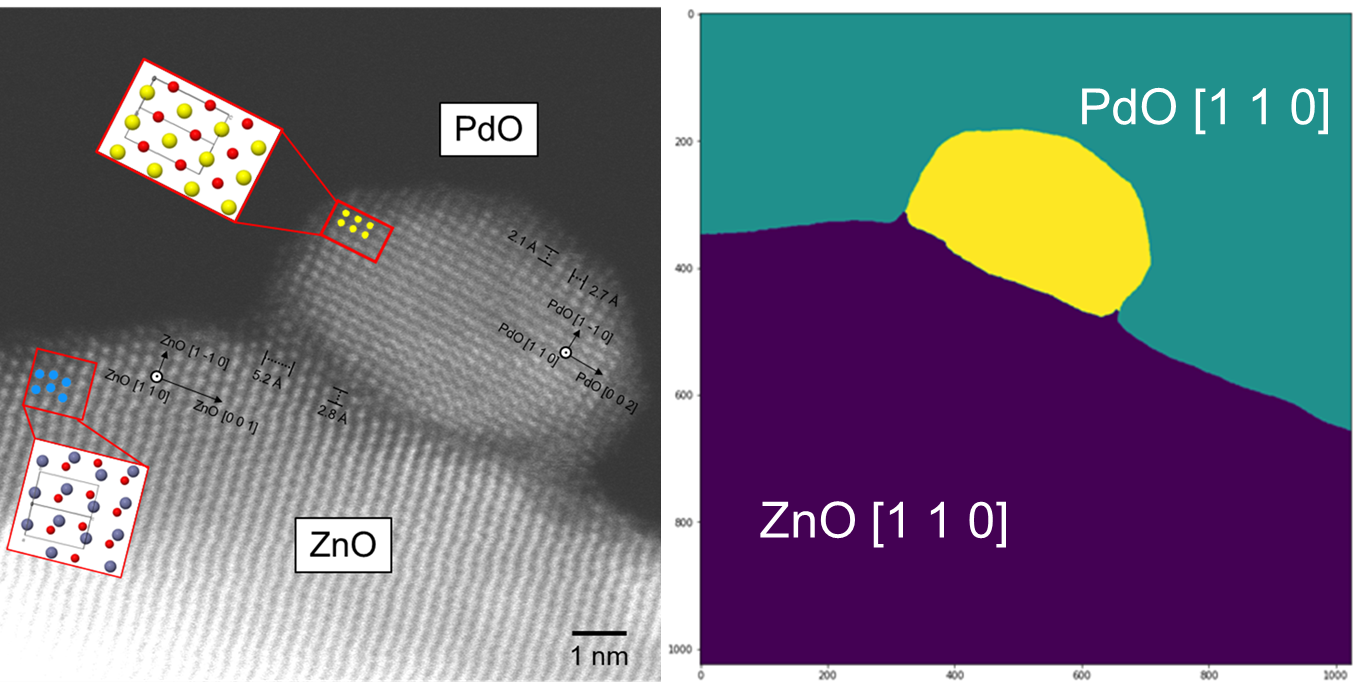


**Figure S1:** Left: DF STEM image of a pristine PdO nanoparticle on ZnO. The PdO nanoparticle is aligned along [110] as shown in the model^[5]^. The ZnO crystal is aligned along [110] as exemplified in the model^[6]^. Pd, Zn and O are represented yellow, blue and red, respectively. Right: Phases of the system are automatically identified by comparing the corresponding FFT and lattice spacings.

After calcination, supported PdO nanoparticles with 5.-wt% Pd loadings were pre-characterised using STEM and XRD. Powder X-ray diffraction was conducted on a Rigaku SmartLab using Cu Kα1 radiation (λ = 1.540598 Å, Ge-Johansson monochromator) within a range of 20 ° < 2θ < 90 °. Acquired SE images, XRD measurements and particle size distributions (PSDs) are shown in **Figure S2**. The SE images indicate a homogenous distribution of nanoparticles on the ZnO surface. Some nanoparticles aggregate to form dense clusters. Next to single, bigger nanoparticles (> 10 nm), many small nanoparticles are present. The density of nanoparticles tends to decrease for lower Pd loadings. Over 100 nanoparticles were measured in two perpendicular directions to determine the PSD of each Pd loading. All PSDs demonstrate that most nanoparticles are smaller than 10 nm with a mean of ~7 nm. With the exception of minor deviations observed in the 5 wt.% loading sample, which exhibits the most compact PSD, the PSDs show a roughly similar trend, indicating that a Pd loading between 1 and 5 wt.% has a minor effect on the particle size. The XRD measurements show similar results for different Pd loadings. For instance, the sample with 5 wt.% Pd loading, which is investigated during reduction, has many characteristic reflections, which can be assigned to ZnO and represent the support. None of the intense peaks correspond to a palladium species. There is a small increase in intensity around 34°, which can potentially be allocated to PdO. The intensity of this hump increases with loading. The 10 wt.% loaded sample was only investigated by XRD not by STEM.


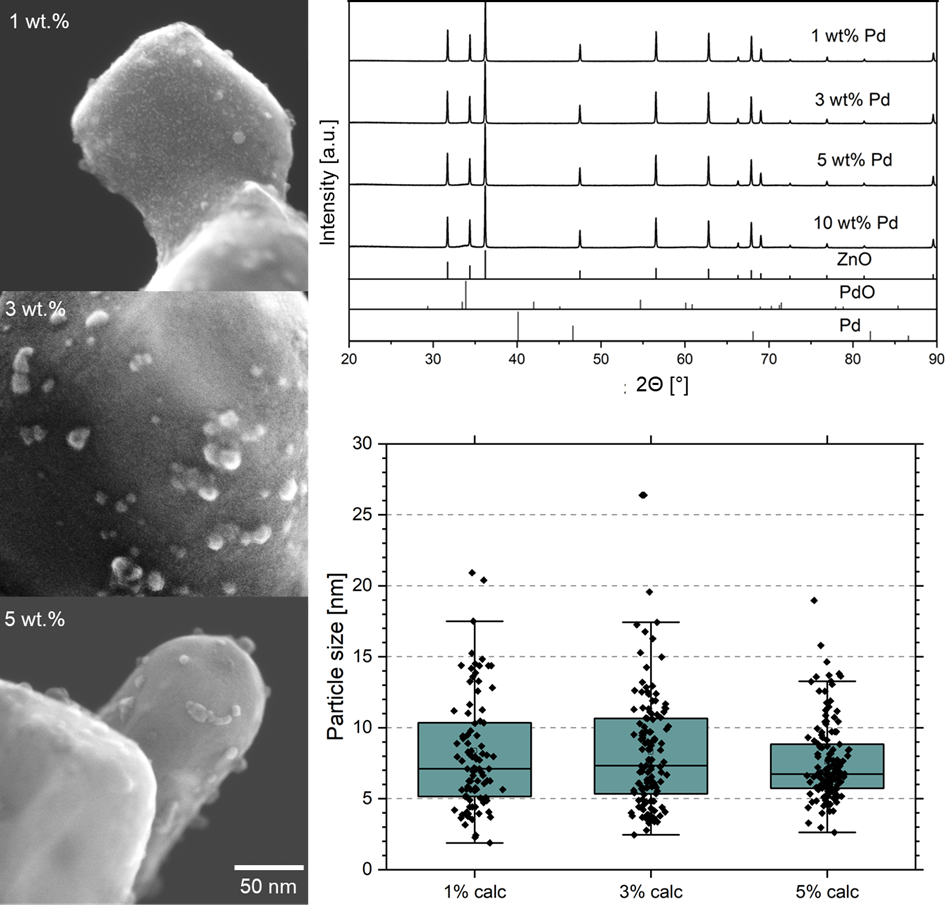


**Figure S2:** Left: Series of SE STEM images of PdO nanoparticles supported on ZnO prepared with different Pd loadings after calcination. Right: Corresponding XRD measurements (top) and particle size distributions (bottom) of the sample with different Pd loadings. XRD reflections of ZnO, PdO and Pd are given as reference. The XRD data of the 5 wt.-% sample shows a small hump around 34°, corresponding to PdO. The strong peaks correspond well to the reference ZnO peaks, identifying the support.

The interatomic spacings are measured by averaging several particle distances along two perpendicular directions. The number of atom rows analysed varies between 3 to 8 depending on the degree of separation. The measured directions $g_{part,\text{ }0min}\text{=}2.1\text{ }Å$ and $h_{part,\text{ }0min}\text{=}2.7\text{ }Å$ are in good agreement with PdO [1 -1 0] $g_{PdO}\text{=}2.15\text{ }Å$ and [0 0 2] $h_{PdO}\text{=}2.68\text{ }Å$.^[61]^ The nanoparticle can accordingly be identified as PdO and is aligned along [1 1 0]. In Figure 1, the Pd atoms are highlighted in yellow to indicate their location within the tetragonal PdO structure model^[5]^. The structure of the support is analysed in the same way. The measured directions $g_{,\text{ }0min}\text{=}2.8\text{ }Å$ and $h_{,\text{ }0min}\text{=}5.2\text{ }Å$ correspond to ZnO [1 -1 0] $g_{ZnO}\text{=}2.82\text{ }Å$ and [0 0 1] $h_{ZnO}\text{=}5.2\text{ }Å$.^[6]^ A ZnO unit cell aligned along [1 1 0] is illustrated by the blue Zn atoms in the ROI in Figure 1. The oxygen atoms represented in red in the structure models for PdO and ZnO are not visible in the DF image.

The crystallographic phase identification confirms the main crystallographic phases of PdO and ZnO, while also indicating the presence of ZnPd crystal phases with different ratios of stoichiometry, which suggests a modulation of the Zn:Pd ratio at the interface during the reaction. Automated geometrical phase analysis suggested a slightly mismatched epitaxial relation between the original ZnO and PdO, probably linked to a morphologically heterogenous substrate.


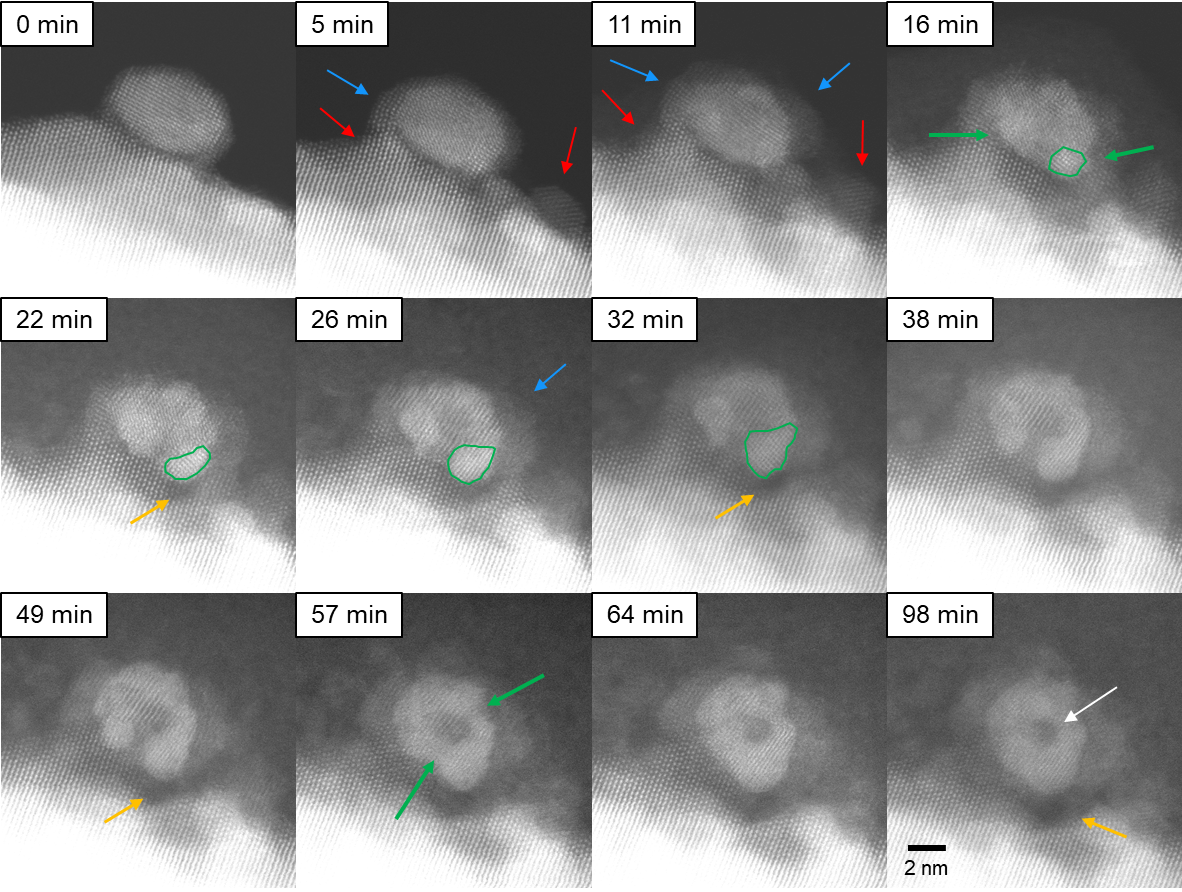


**Figure S3:** Full Series of DF STEM images of the evolving nanoparticle-support structure during reduction in a hydrogen atmosphere at room temperature, corresponding to the images shown in Figure 1.


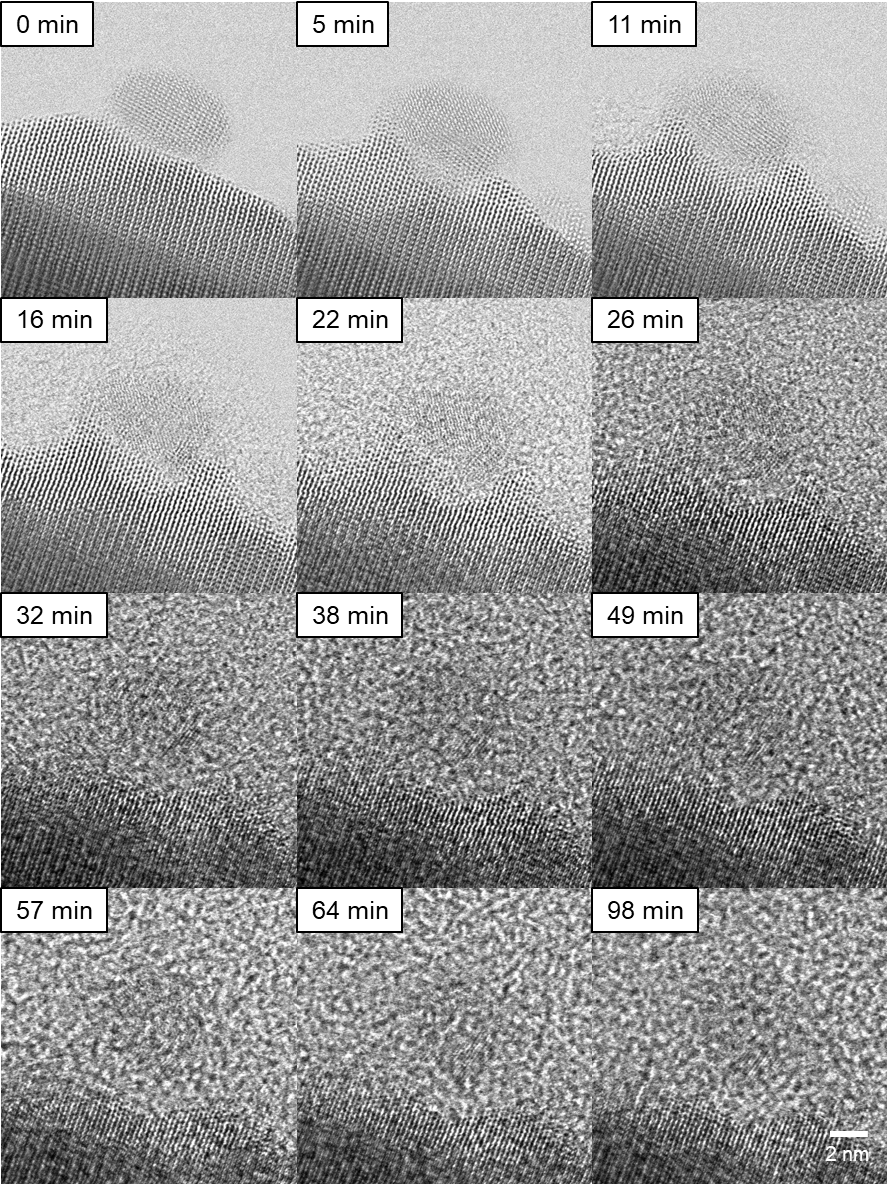


**Figure S4:** Series of BF STEM images of the evolving nanoparticle-support structure during reduction in a hydrogen atmosphere at room temperature. Corresponding DF STEM images are shown in Figure 1.

**Figure S5:** Phase analysis of dark field images from Figure 1 during *in-situ* structure transformation using FFTs of the areas marked by rectangles. In the beginning (0 min) a PdO particle on ZnO support observed. The second row (16 min) shows the presence of Pd region at the bottom of the particle. At 32 min, the identification of a phase becomes somewhat ambiguous. A best match is found with the Pd_2_Zn Pnma phase zone axis (621). The observed angle of 79º between planes however refers to a heavily strained cubic or a tetragonal/orthorhombic phase. It is hypothesized that Pd incorporates Zn progressively deforming the cubic unit cell into an orthorhombic phase that might explain the angle of 79º between the planes in the second image.


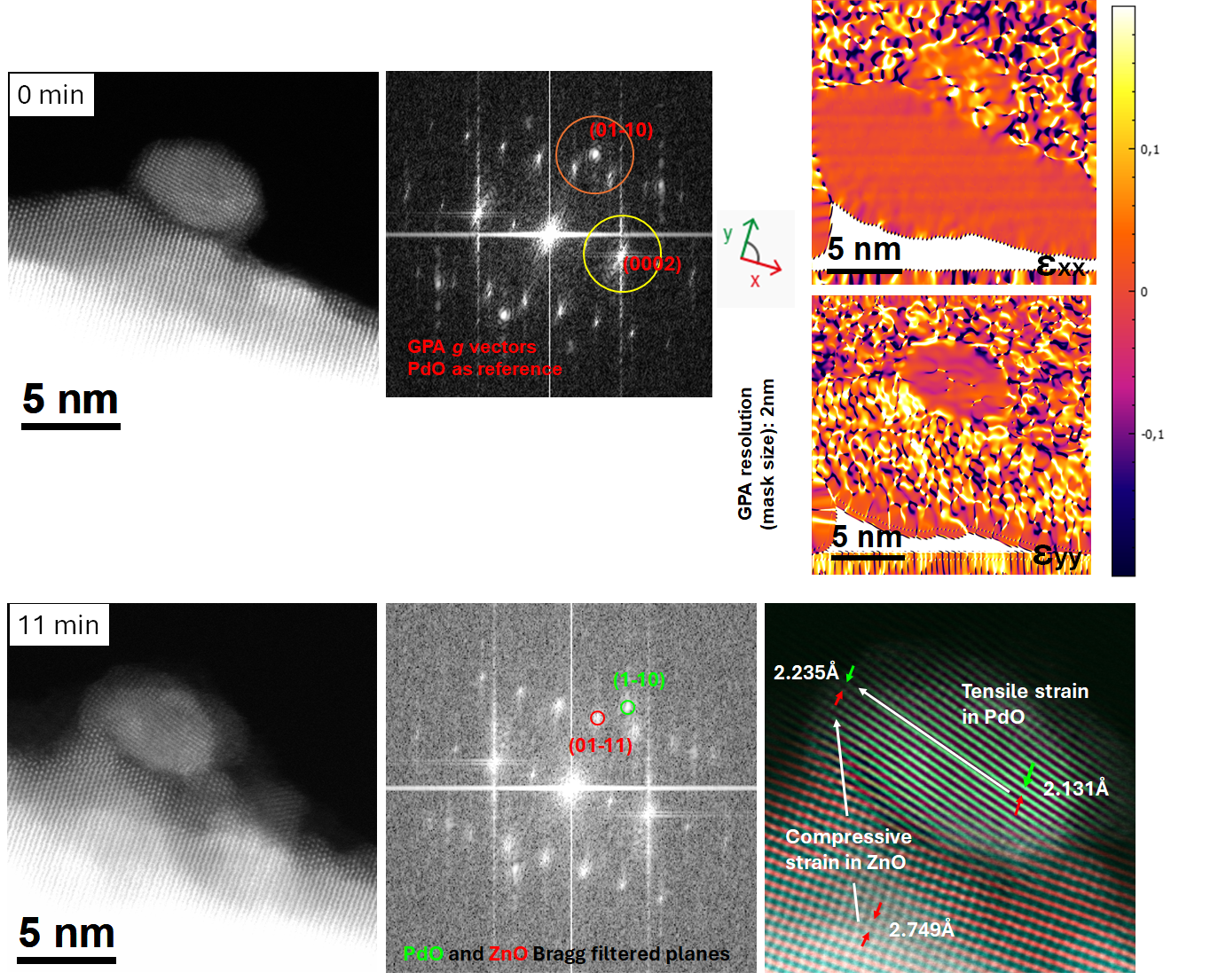


**Figure S6:** Upper row, 0 min: geometric phase analysis (GPA) of the PdO particle. Mismatch of the (002) planes involved between the PdO and ZnO lattice: (2.665 Å-2.603 Å) / 2.603Å = 2.38%. The average dilatation extracted from the εxx relative strain map is close to 0 %. The epitaxial strain from the ZnO (0002) and PdO (002) lattice yields -2.3 % compressive strain for the PdO vertical planes while the horizontal planes are relaxed. This elastic strain component minimises the interface misfit and explains the observed close 9º rotation of the growth planes (1-10).
Lower row, 11 min: The overgrowth of the ZnO on the PdO particle induces strain in the lattices. The substrate presents relaxed (01-10) planes that are compressed up to a -18.7%, while the PdO relaxed planes experience tensile strain at the core-shell interface of 4.9%. These high strain values may indicate that the transformation toward the intermetallic ZnPd phase may occurs via highly strained continuous alloying and not via the formation of intermediate intermetallic equilibrium phases.


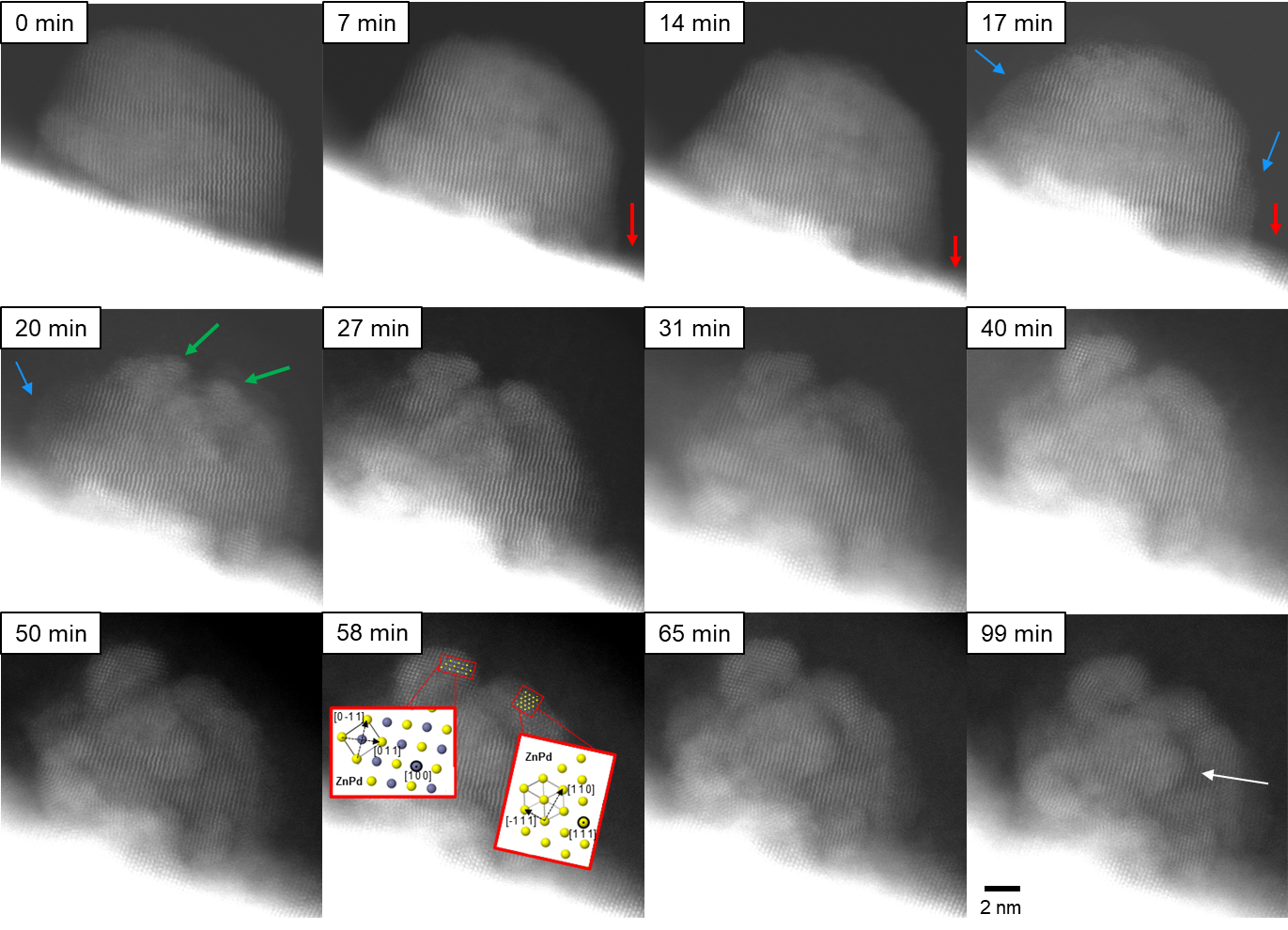


**Figure S7:** Full Series of DF STEM images of the evolving nanoparticle-support structure during reduction in a hydrogen atmosphere at room temperature, corresponding to the images shown in Figure 1.


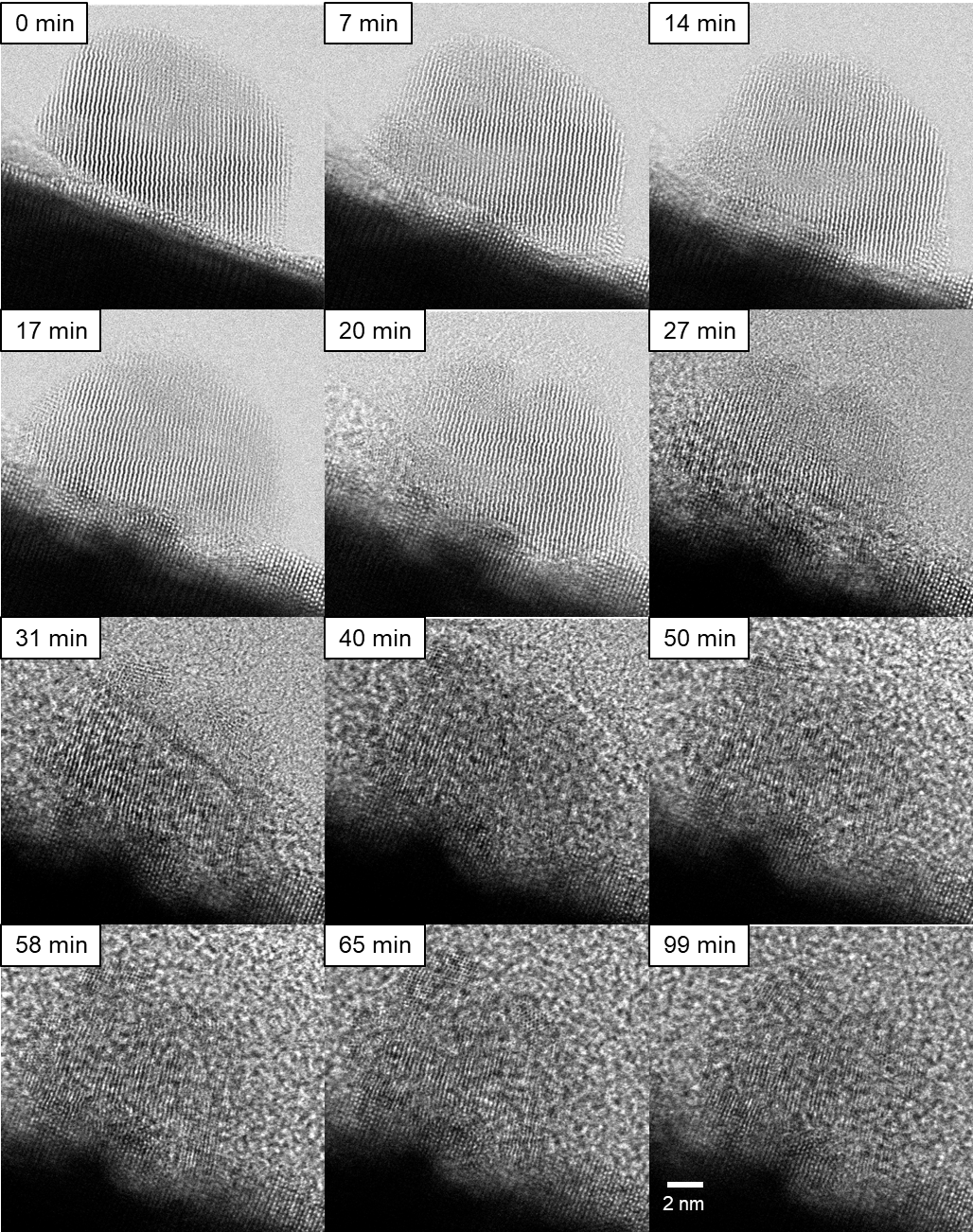


**Figure S8:** Series of BF STEM images of the evolving nanoparticle-support structure during reduction in a hydrogen atmosphere at room temperature. Corresponding DF images are shown in Figure 2.


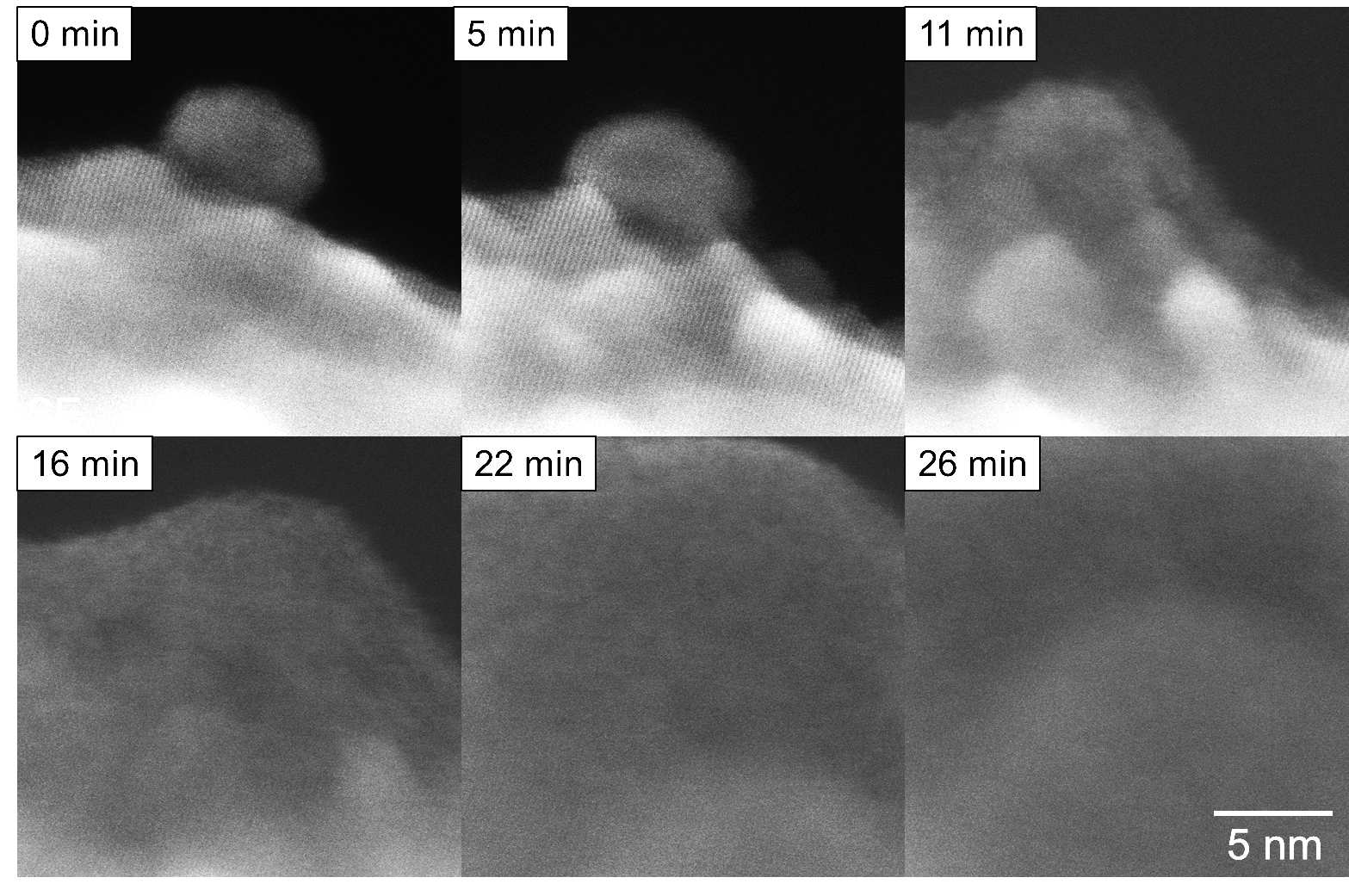


**Figure S9:** Series of SE STEM images that show formation of contamination over time during reduction in a hydrogen atmosphere at room temperature. Specimen structures cannot be resolved after 22 min.

Formation of surface contamination is observed after 11 min. Since the detection of SE is surface sensitive, SE imaging is particularly affected by an increase in contamination over time. Sample areas, which are imaged with SE longer than 11 minutes after the start of reduction, are largely covered by contamination. **Figure S9** demonstrates the degree of contamination.


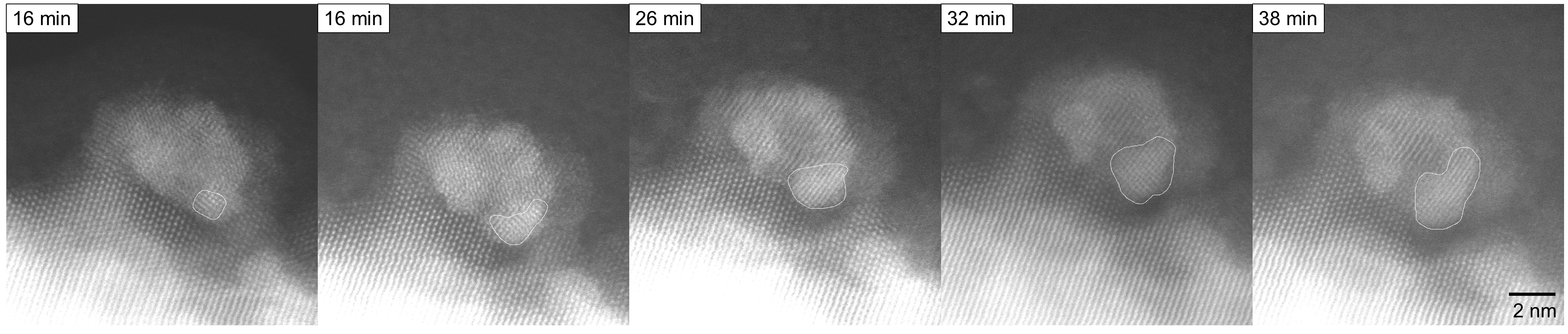


**Figure S10:** Series of DF STEM images illustrating the phase growth during reduction. The time stamps identify time of reduction. The corresponding measurements are displayed in Figure 3.


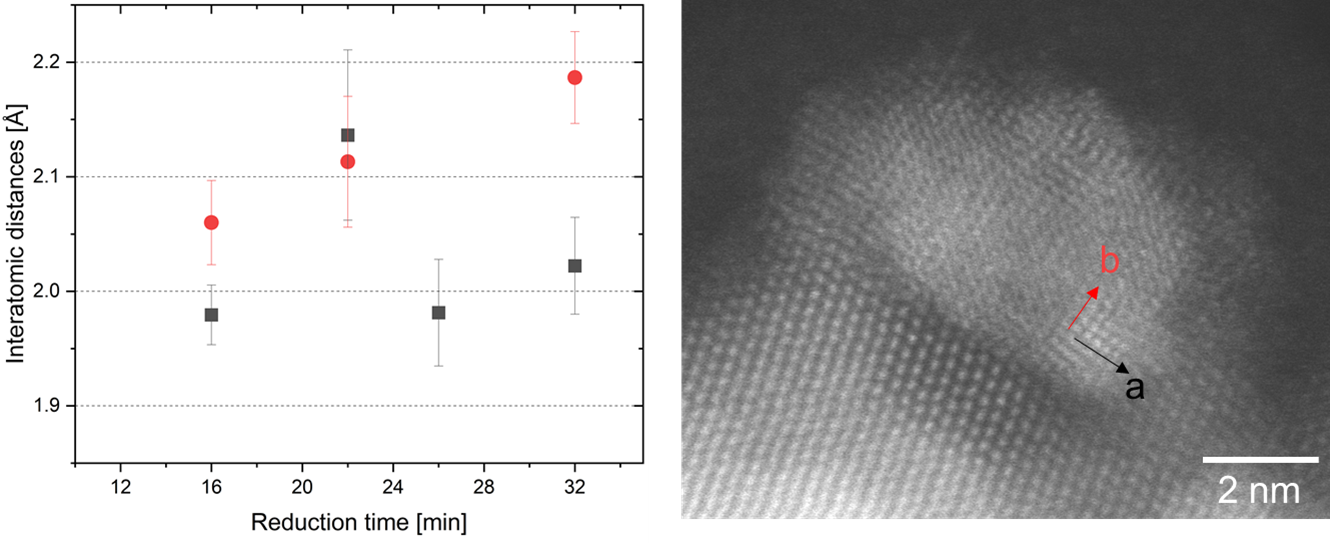


**Figure S11:** Measurement of the interatomic distances of the phase forming at the particle along the displayed directions between 16 and 32 min of reduction using DF STEM imaging. Direction a is shown in black, direction b in red. The resolution of the spacing in direction b was insufficient to measure the lattice spacings after 22 min.


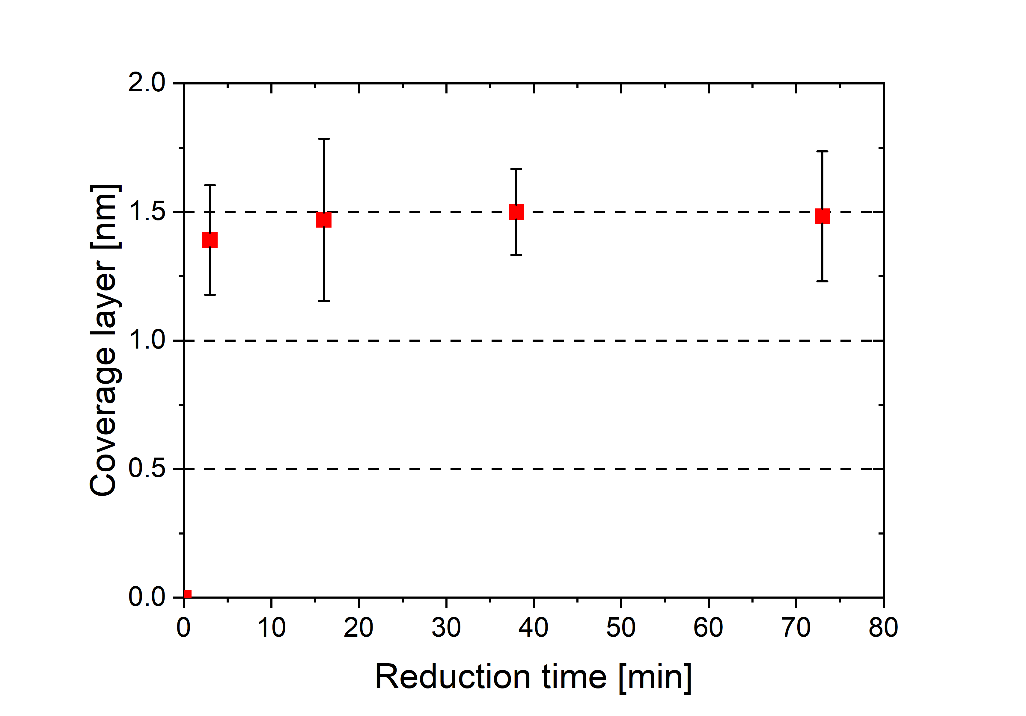


**Figure S12:** The change in thickness of the coverage layer is shown as a function of reduction time. The coverage layer converges to a thickness of ~1.5 nm, after it is formed. The corresponding covered nanoparticle is displayed in Figure 1. At each reduction time, the layer thickness was measured at 9 different locations and averaged.


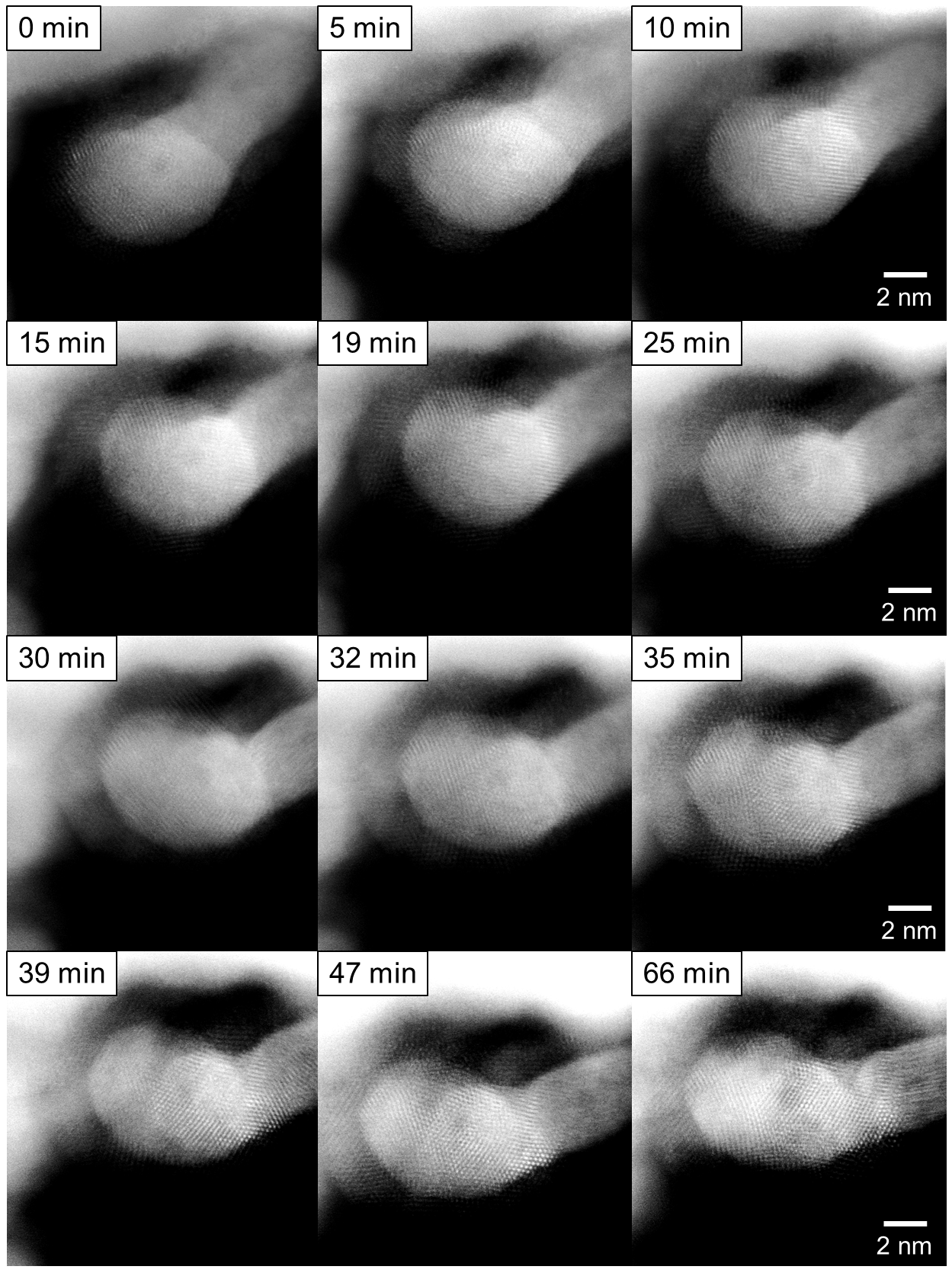


**Figure S13:** Series of DF STEM images during reduction of PdO at room temperature after a pre-treatment at 200 °C. Time stamp indicates the reduction time. ZnO is activated within the first 5 min. After initial nanoparticle support interaction, structural changes of the nanoparticle are observed.

**Characterisation of Pristine Palladium Oxide Nanoparticles**

In another experiment, PdO supported on ZnO was annealed in vacuum at 200 °C for 60 min prior to reduction to remove residual hydrocarbons from the sample. The sample was then reduced under the same conditions as described above. **Figure S13** shows the sample during reduction at room temperature after a preheating treatment. Here, ZnO is activated first as during the previously described experiment. Small clusters are formed on the ZnO surface, which preferentially migrate to the nanoparticle. When the nanoparticle is decorated by a ZnO layer, the nanoparticle is transformed by a solid state reaction. Intriguingly, the transformation is activated at the interface first and proceeds through the crystal. During reduction, the nanoparticle tends to grow in size. Quantification of the particle growth is difficult due to a lack of segmentation. In contrast to the experiment with no preheating, no contamination is formed during beam exposure.

Surface contaminations are formed in the vicinity of the nanoparticles during reduction. The present contamination could be water and formed by recombination of activated hydrogen with oxygen provided by the oxides. However, as contamination could not be found homogenously over the specimen and at the sample which were annealed prior to the reaction, the sample is more likely to be contaminated by residual hydrocarbons, which are absorbed during preparation and are activated by the electron beam.

**Evaluation of Electron Beam Effects During Reduction**

Nanoscale structures are vulnerable to high energetic electron beam exposure. The nature of such beam effects might differ strongly depending on the energy and intensity of the electron beam and on the properties of the exposed material. In particular, during *in-situ* experiments, where structural changes activated by the imposed conditions are anticipated, it is important to assess the effect of the electron beam on the material under investigation. In addition, the electron beam could also interact with the present atmosphere, forming a plasma and change the reaction conditions.

In order to evaluate the electron beam effect on the PdO nanoparticles, we have exposed PdO particles with the same electron dose as in the reduction experiments (**Figure 1 and 4**) in a series of 12 consecutive image acquisitions at ambient temperature in vacuum (**Figure S14**). This experiment shows evidence of structural transformations due to electron beam irradiation, for example at the lower right part of the left nanoparticle (see arrow in **Figure S14**) demonstrating that the influence of the electron beam during the reduction experiments cannot be neglected. We note the waiting time between individual image acquisitions at the end of the experiment in **Figure S14** has been shortened compared to the gas experiments (**Figures 1 and 4**) because structural changes without an electron beam in a vacuum can be ruled out. **Figure S15** shows an EDX map of the respective area.

Furthermore, we have analysed reference sample locations, which were not exposed to the electron beam during the *in-situ* experiment. Such images, which are acquired under identical conditions to the *in-situ* experiment presented in our manuscript, are displayed in **Figure S16**. During the *in-situ* experiments, the electron beam was only used to capture the individual images and to focus (approx. 1 min each) on the corresponding sample site. During the main part of the *in-situ* experiment, the electron beam was either used to image another sample area or for most of the time not present by closing the gun valve.


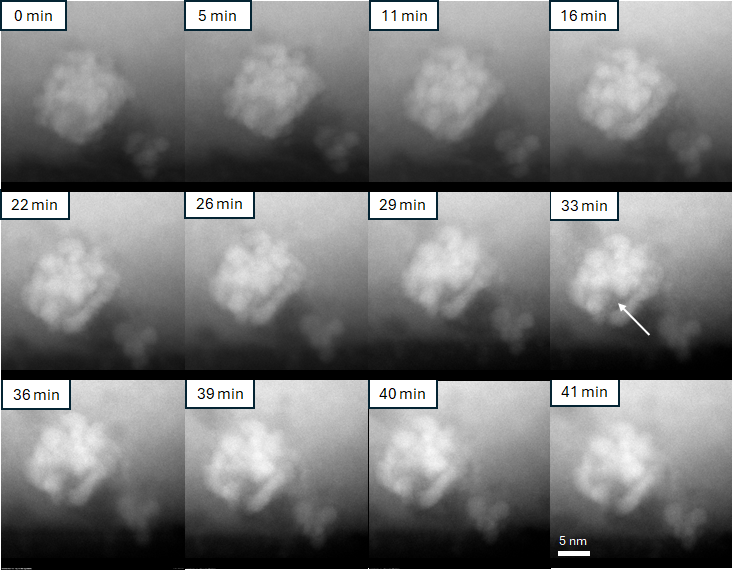


**Figure S14:** DF STEM image series of a supported PdO nanoparticles in vacuum at ambient temperature using the same electron dose as in the reduction experiments (Figure 1 and 4). Slight structural transformations (for instance indicated by the arrow) are present.


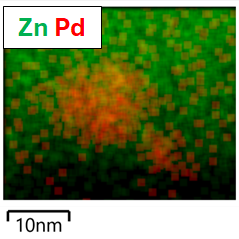


**Figure S15:** STEM-EDX map of the particles imaged in Figure S14.


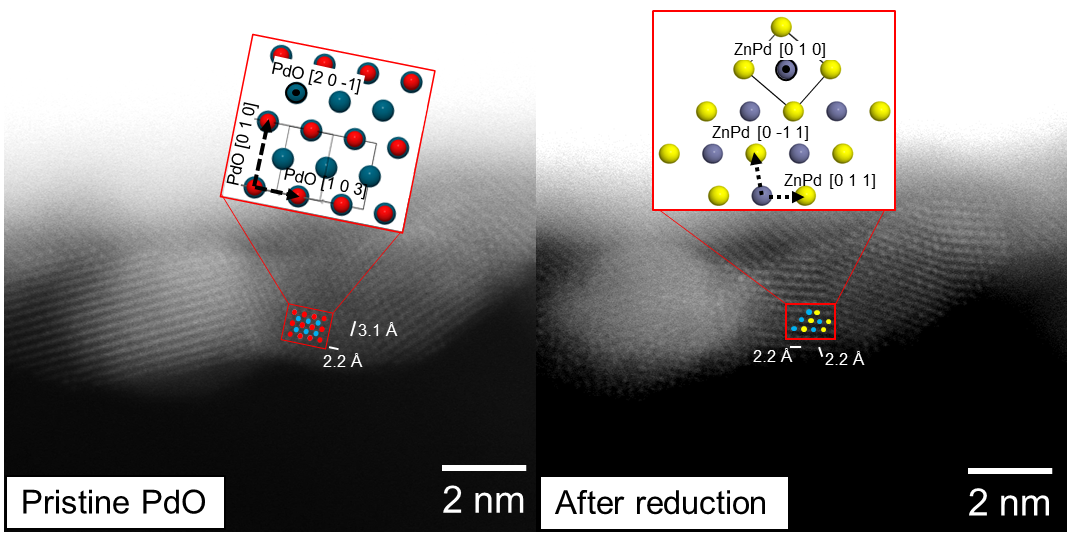


**Figure S16:** DF STEM images of a supported nanoparticle before and after reduction in a hydrogen atmosphere at room temperature. The location was not exposed to the electron beam in presence of a hydrogen atmosphere. While the pristine nanoparticle matches with the lattice of PdO along [2 0 -1], the nanoparticle after reaction corresponds well to ZnPd along [0 1 0]. Zn is coloured in blue, Pd in yellow, O in red. The structure models are taken from literature.^[6,7]^

The crystal structure of the nanoparticle before reduction can be identified as PdO. The lattice spacings measured of 3.1 ± 0.1 Å and 2.2 ± 0.1 Å are in good agreement with PdO (0 1 0) of 3.03 Å and (1 0 3) of 2.14 Å. After reduction, the crystal structure of the nanoparticle has changed. The measured lattice spacings of 2.2 ± 0.1 Å in each direction match best with ZnPd (0 1 1) of 2.18 Å, indicating the formation of an intermetallic compound. Although the structure of the nanoparticle after reduction is largely single-crystalline, some regions, e.g. the left edge, have an irregular morphology. In addition, the activation of the support is weaker than that of the support, which was exposed to the electron beam *in-situ*. Less encapsulation by the metal oxide is visible.

**Reduction of Palladium Oxide at 200 °C**

In addition to the reduction experiments performed at room temperature, supported PdO was reduced *in-situ* at 200 °C. The sample was tempered at 200 °C for 20 min, before hydrogen was injected. **Figure S17** and **Figure S18** show the structural evolution of the system during reduction at two different magnifications. The pristine crystal structure of the support and nanoparticle with 2.4 ± 0.1 Å and 2.1 ± 0.1 Å matches well with ZnO (0 1 1)^[5]^ of 2.48 Å and PdO (1 1 0)^[6]^ of 2.15 Å. ZnO has an undistorted morphology and is only in contact with the nanoparticles on the supporting side. After 3 min of reduction, the support evolves and forms small ZnO cluster on the support surface. The nanoparticles are encapsulated by a continuous ZnO layer, as shown by the green shape in **Figure S17**. The perimeter of the nanoparticle is marked in red as guide to the eye. The thickness of the coverage layer is ~1.5 nm, as shown in **Figure S17**. After the initial encapsulation, the support undergoes further morphological changes, as displayed by the red arrows **Figure S18**. The evolution includes the formation of spherical ZnO enrichments, which grow with time in contrast to the encapsulation layer. Near the interface of the nanoparticle and the support, ZnO is removed and forms a small neck underneath the nanoparticle, as highlighted by the blue dotted lines. Given that other features of the ROI in **Figure S18** are immobile, it can be concluded that the neck does not grow outwards, but that ZnO dissolves around the interface. The phenomenon occurs simultaneously with the encapsulation of the nanoparticle.


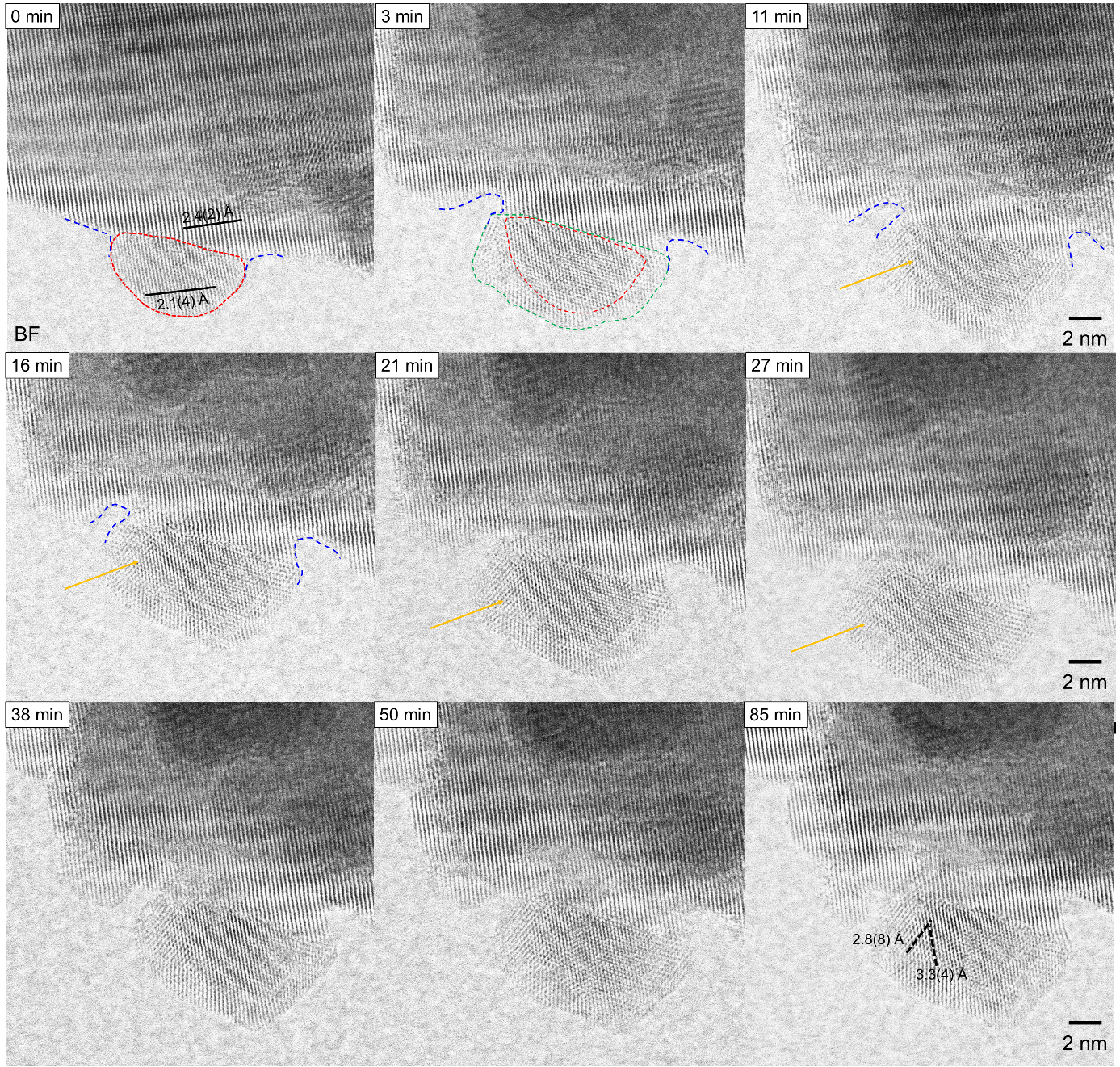


**Figure S17:** Series of BF STEM images shown during the reduction of supported PdO at 200 °C. The time of hydrogen injection is displayed. The region marked in red highlights the nanoparticle, the region marked in green the encapsulation by ZnO. Neck formation and ZnO dissolution is marked in blue. The orange arrows indicate a phase transformation.

The encapsulation has no influence on the size of the nanoparticles. However, a change in the crystal structure can be recognised. The crystal structure is single-crystalline throughout the nanoparticle. The lattice parameters in three different directions (a, b, c) are measured during reduction, as shown in **Figure S19**. Since these lattice parameters are the same on the right side of the nanoparticle within the measurement accuracy, it can be concluded that the same crystal structure is present between 3 and 85 minutes of reduction. Assuming no change in crystal structure after 3 min of reduction, a representative identification of the crystal structure is performed after 16 minutes. As defined in **Figure S19**, the interatomic spacings measured along a, b and c are 4.1 ± 0.1 Å, 2.5 ± 0.1 Å and 2.3 ± 0.1 Å. These values are in good agreement to Pd^[9]^ along [1 1 0] and ZnPd^[7]^ along [1 1 1]. Thus, a may correspond to Pd (0 0 1) of 3.89 Å or ZnPd (1 1 0) of 4.09 Å, b to Pd (-1 1 0) of 2.75 Å or ZnPd (-1 1 1) of 2.64 Å and c to Pd (-1 1 2) of 2.38 Å or ZnPd (3 1 1) of 2.44 Å. The measured angles of 63.5 ± 0.8° and 58.9 ± 2.0° are in between the theoretical values for Pd of 54.7° and 65.3° and for ZnPd of 60°. In addition, the large standard deviation underlines that manual angle measurement is error-prone. A distinct allocation for the crystal structure of the right side of the nanoparticle to ZnPd or Pd is therefore not feasible. In contrast to the right side, on the left side of the particle, as indicated by the orange arrows in **Figure S17**, the phase transforms with time. After 85 min, the measured lattice spacings of 2.9 ± 0.1 Å and 3.3 ± 0.1 Å match to ZnPd (0 0 1) of 3.27 Å and (1 0 0) of 2.89 Å, allowing an allocation to ZnPd.


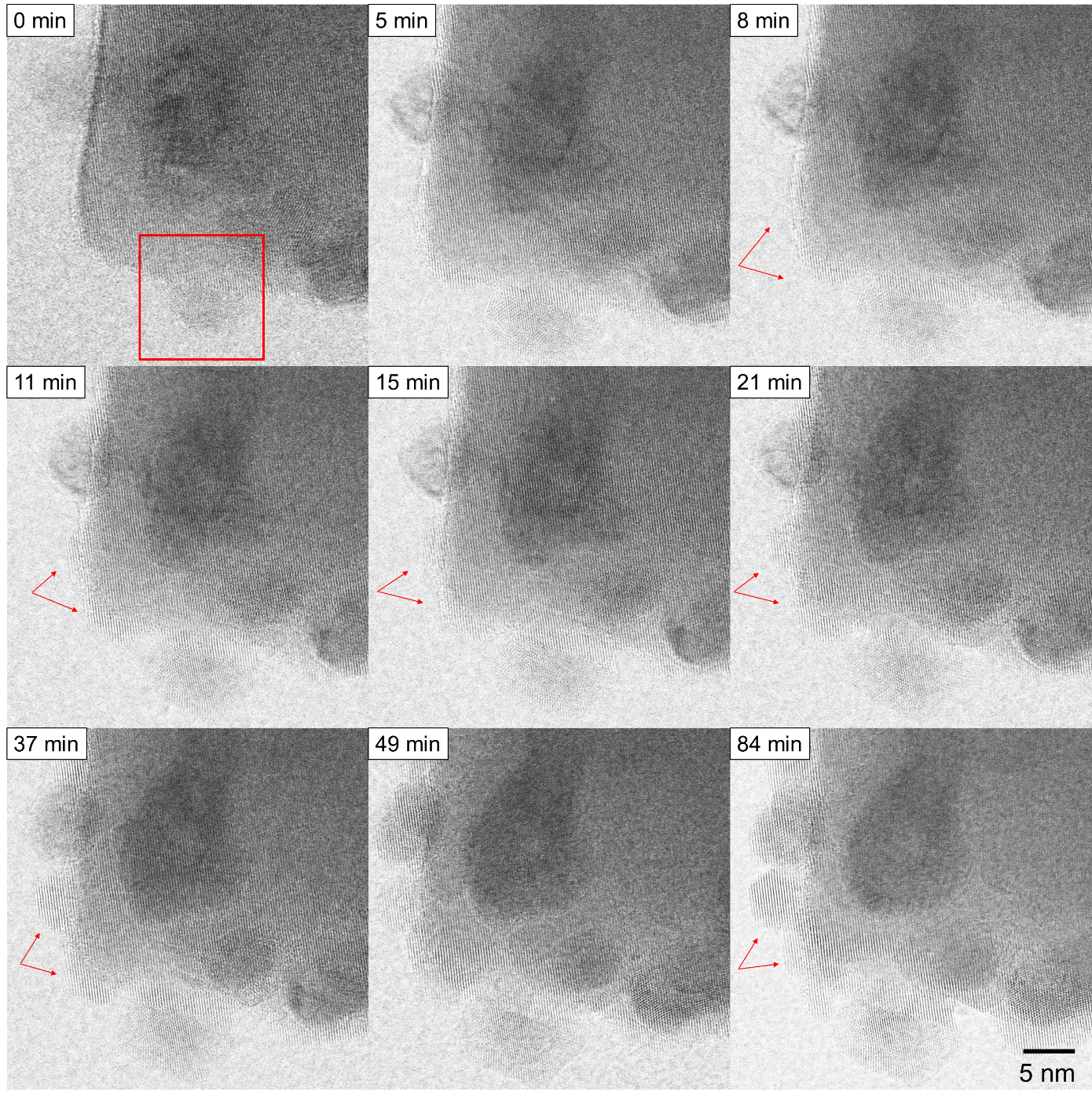


**Figure S18:** Series of BF STEM images during the reduction of supported PdO at 200 °C. The time of hydrogen injection is displayed. Red ROI marks location shown in **Figure S17**. The support is activated and encapsulates the nanoparticles. Red arrows show morphological evolution of ZnO.


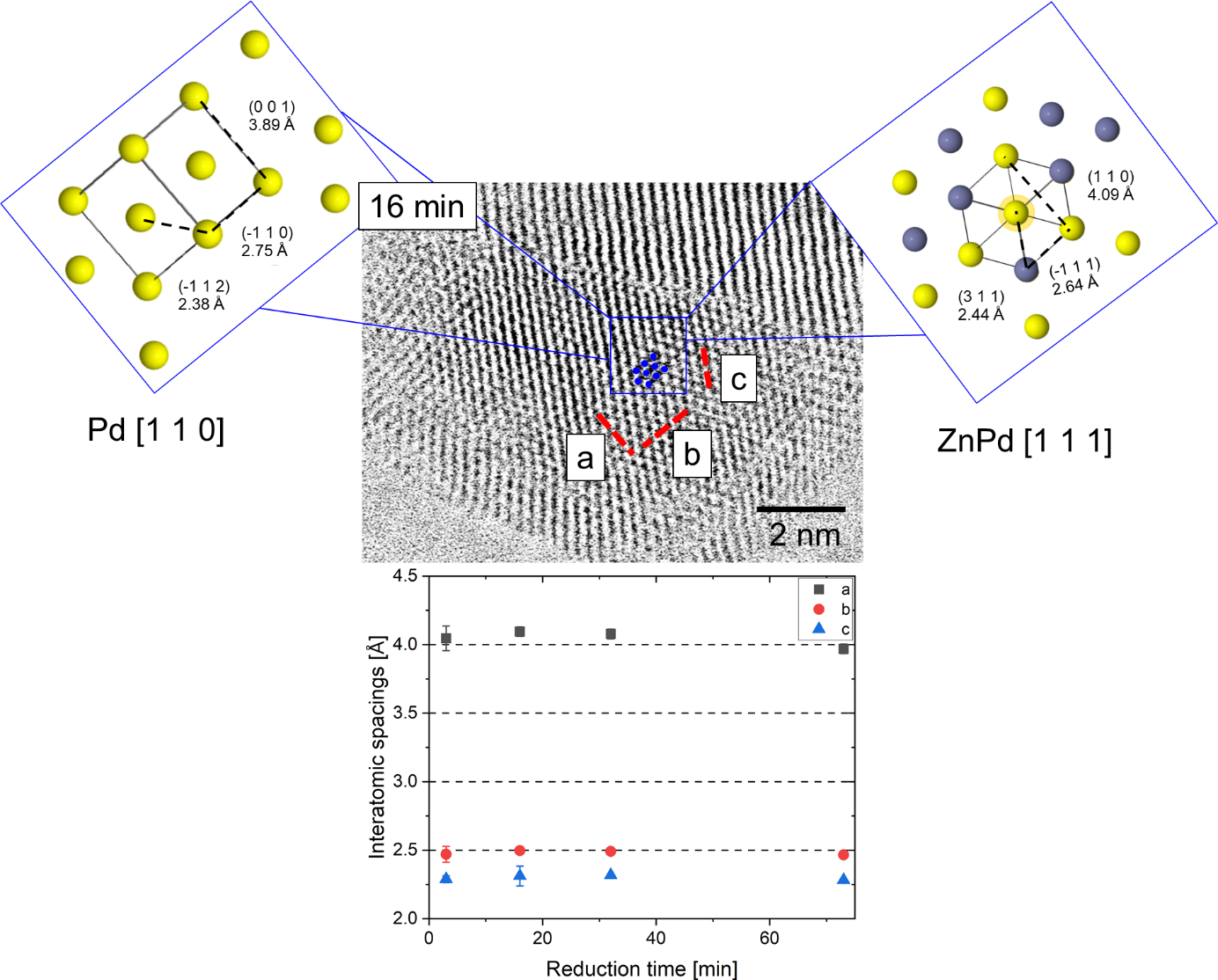


**Figure S19:** Top: BF STEM image of a nanoparticle shown after 16 min of reduction. The structural evolution during reduction is illustrated in **Figure S17**. The crystal structure of the nanoparticle may correspond to Pd along [1 1 0] or ZnPd along [1 1 1], since the interatomic spacing and angles match well to the models taken from references.^[7,9]^ Bottom: The measured interatomic spacings along a (black square), b (red circle) and c (blue triangle) direction displayed over the past reduction time. The spacings show no considerable change.

**Evaluation of Beam Effects During Reduction at 200 °C**

**Figure S20** shows sample locations, which have been imaged before and after, but not during, reduction at 200 °C to evaluate the effect of the electron beam. The crystal structure of the nanoparticle displayed in the left images can be identified as Pd along [1 1 0]. Intriguingly, the Pd phase is already present at 200 °C in vacuum before hydrogen is injected. After reduction, the lattice shows mild distortions, but can still be assigned to Pd.


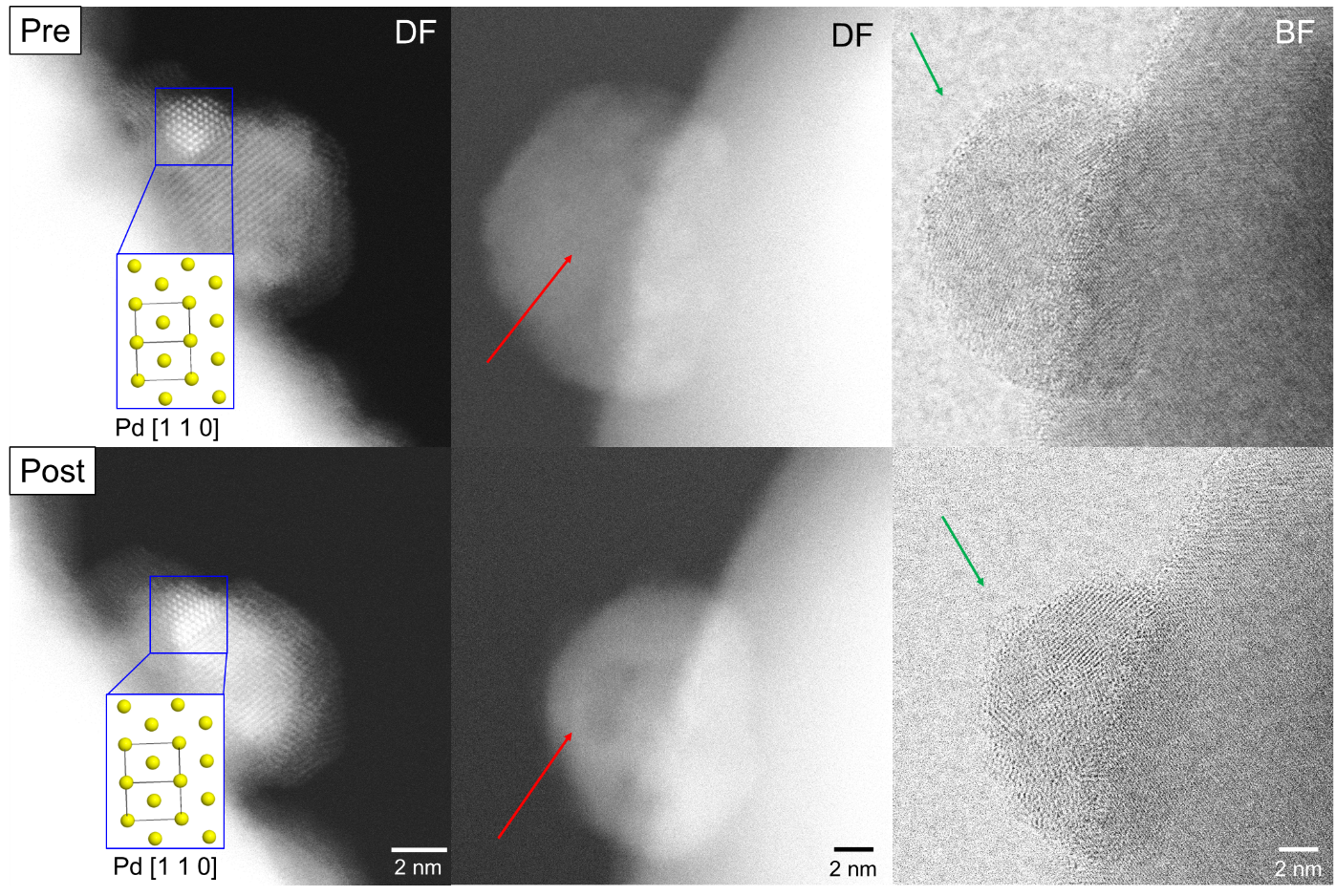


**Figure S20:** DF and BF STEM images showing a comparison between sample locations before (top) and after (bottom) reduction at 200 °C. Left: Pd nanoparticle exhibit the same crystal structure along [1 1 0] before and after the reaction. Middle and Right: Identical sample location imaged in DF and BF. The red arrows show the formation of regions with darker DF contrast, indicating a mass loss. The green arrows indicate the nanoparticle decoration by the oxide.


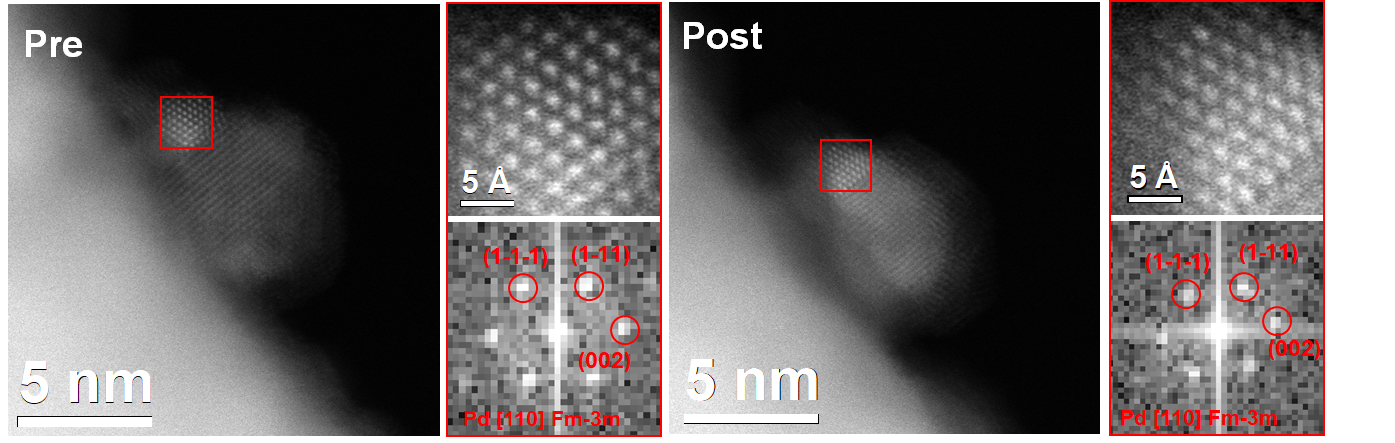


**Figure S21:** FFT analysis of the DF STEM images in Figure S18 showing the local presence of Pd phase.

In vicinity of the nanoparticle, the support is activated and has formed a cluster at the surface. Additional support activity can be seen on the nanoparticle, which is displayed in DF and BF images in **Figure S20**. Here, the BF image reveals formation of ZnO decorations on the nanoparticle, as marked by the green arrows. In the DF image, the nanoparticle is equally bright before reduction, indicating a homogenous thickness and mass distribution. After reduction, the edge appears brighter than the core, forming a core-shell structure, as highlighted by the red arrows.

**Effects Induced by the Experimental Conditions**

An electron beam introduces kinetic energy and charge to the observed system. In case of a *in-situ* gas experiment, the properties affect the sample and environment. Since the electron beam probe is in the nm-range, a beam effect will only be anticipated in imaged regions. Interaction between the electron beam and hydrogen may form hydrogen plasma. Due to the presence of atomic, ionic and excited hydrogen species, hydrogen plasma is recognised to exhibit a high reduction capacity for many metal oxides even at low temperature, typically superior to that of molecular hydrogen.^[10]^ A quantification of the reduction potential of different hydrogen species for metal oxides is shown in Ellingham diagrams. Such diagrams show that the Gibbs free energy change for atomic and ionic hydrogen is 3 and 15 times less than that of molecular hydrogen, respectively, confirming that activated hydrogen is capable of directly reducing ZnO if present.^[10,11]^ A reduction of sole ZnO under identical imaging and environmental conditions were not found. Consequently, it can be concluded that the amount of hydrogen plasma generated by the electron beam is only small and its potential effect can be neglected for *in-situ* experiments.

Direct interaction between the electron beam and the specimen must be considered, as nanoparticles can suffer from beam effects such as structural and electronic changes as well as local heating effects. Kim et al. demonstrated in their work that strong electron beam irradiation with beam energies of 1 MeV induces structural defects to Pd-supporting ZnO.^[12]^ Typical structural defects, which can be generated^[13]^, are oxygen vacancies and Zn interstitial defects (Frenkel defects^[14]^). These Zn interstitial defects may donate additional delocalized electrons to the system that may lower the work function of ZnO and increasing the electron depletion zone in ZnO. In addition, donor defects in semiconductors can act as active sites in the chemisorption process^[15-17]^, thereby amplifying the process itself. It is likely that such phenomena also apply during support activation of the present *in-situ* reduction experiments, so that electron transfer to the nanoparticle is increased and the above-mentioned mechanism of ZnO migration is facilitated. Sample regions, which were exposed to the electron beam during reduction, exhibit more pronounced support activity than reference regions. The amplified support activity is reflected in the enhanced encapsulation and the formation of ZnO clusters on the surface. An enhancement in chemisorption capabilities is likely to participate in the support activation, since the support was not activated by pure beam exposure but in combination with hydrogen during *in-situ* experiments. However, the imaging conditions during the reduction experiment result in a lower beam dose to the system than the intentional electron beam treatment by Kim *et al.*^[12]^ with lower exposure times (20 s instead of 54 s), lower beam energy (200 kV instead of 1000 kV) and lower beam current (10 µA instead of 1000 µA), so that a lower beam effect on the present *in-situ* experiment can be expected. Since both mechanisms were also found at reference locations with no beam exposure during reduction, the mechanisms were facilitated but not triggered by the electron beam. At the same time, these findings underline that SMSI and RMSI can be facilitated and potentially activated by an electron beam. This activation mechanism is interesting for the catalyst design of supported nanoparticles, where metal-support interactions enhance catalytic properties and therefore are deliberately triggered.

In addition to the energy of the electron beam, charges may accumulate on the support due to low electric conductivity of ZnO, manipulating the electric properties of ZnO and thus migration of the Zn/O atoms. So far, the debates on the nature of the charge induced by the electron beam in TEM have not yet been resolved^[18,19]^, so that no assessment of the effects of the potential charge on the reduction will be made at this point.

Interaction with the electron beam can deposit energy to the specimen, generating heat and increasing the temperature of the system. The extent to which the local temperature is changed by the electron beam depends on numerous parameters such as the heat capacity and conduction and size of the materials, the electron beam dose and energy, the proportion of elastic and inelastic interaction between the sample and the beam, so that precise quantification is difficult and depends sensitively on the material and imaging system used.^[20,21]^ As a detailed quantification of these processes would exceed the scope of this work, the temperature will be estimated at this point using literature values. The temperature gain of materials in the nanometre range is normally in the two-digit Kelvin range and increases with increasing particle size and beam current and decreasing with higher accelerating voltages.^[22]^ Therefore, an increase in temperature (< 50 °C) is expected in the present *in-situ* experiment, which introduces minor changes only. For the room temperature experiments, the temperature increase caused by the electron beam may facilitate the observed structural changes due to higher diffusion.

Gas *in-situ* experiments in an open cell are limited to relatively low pressure. In the present E-STEM experiment the hydrogen pressure applied was around 1.5 Pa, which is significantly lower than the partial pressure of 100 kPa in the reduction by Penner *et al*^[23]^ and the *ex situ* investigation of the sample. The reaction kinetics of chemical reactions are directly determined by the present chemical potential and thus the partial pressure.^[24,25]^ A low partial pressure can therefore change the kinetics and the outcome of a reaction and also applies during synthesis. Such a pressure-related discrepancy in reaction kinetics is referred to as a ‘pressure gap’ and must be considered here. The partial pressure of hydrogen in an atmosphere therefore determines its reducing properties. A higher hydrogen content increases the reducing properties.

References

[1] N. Iwasa, S. Masuda, N. Ogawa, N. Takezawa, Steam reforming of methanol over Pd/ZnO: Effect of the formation of PdZn alloys upon the reaction *Appl. Catal. A, Gen.* **1995**, *125*, 145–157.

[2] R. F. Egerton, Dose measurement in the TEM and STEM *Ultramicroscopy* **2021**, *229*, DOI 10.1016/j.ultramic.2021.113363.

[3] M. Botifoll, I. Pinto-Huguet, E. Rotunno, T. Galvani, C. Coll, P. H. Kavkani, M. C. Spadaro, Y.-M. Niquet, M. B. Eriksen, S. Martí-Sánchez, G. Katsaros, G. Scappucci, P. Krogstrup, G. Isella, A. Cabot, G. Merino, P. Ordejón, S. Roche, V. Grillo, J. Arbiol, Artificial Intelligence End-to-End Workflow for Transmission Electron Microscopy: From Data Analysis Automation to Materials Knowledge Unveiling **2024**, 1–43.

[4] M. Botifoll, I. Pinto-Huguet, J. Arbiol, Machine learning in electron microscopy for advanced nanocharacterization: current developments, available tools and future outlook *Nanoscale Horizons* **2022**, *7*, 1427–1477.

[5] V. Y. Zenou, D. E. Fowler, R. Gautier, S. A. Barnett, K. R. Poeppelmeier, L. D. Marks, Redox and phase behavior of Pd-substituted (La,Sr)CrO3 perovskite solid oxide fuel cell anodes *Solid State Ionics* **2016**, *296*, 90–105.

[6] K. Park, D. A. Hakeem, J. W. Pi, G. W. Jung, Emission enhancement of Eu3+-doped ZnO by adding charge compensators *J. Alloys Compd.* **2019**, *772*, 1040–1051.

[7] E. J. Peterson, B. Halevi, B. Kiefer, M. N. Spilde, A. K. Datye, J. Peterson, L. Daemen, A. Llobet, H. Nakotte, Aerosol synthesis and Rietveld analysis of tetragonal (β1) PdZn *J. Alloys Compd.* **2011**, *509*, 1463–1470.

[8] O. Glemser, G. Peuschel, Beitrag zur Kenntnis des Systems PdO/H 2 O *Zeitschrift für Anorg. und Allg. Chemie* **1955**, *281*, 44–53.

[9] O. Loebich, C. J. Raub, Das zustandsdiagramm lithium-palladium und die magnetischen eigenschaften der Li-Pd-Legierungen *J. Less Common Met.* **1977**, *55*, 67–76.

[10] K. C. Sabat, P. Rajput, R. K. Paramguru, B. Bhoi, B. K. Mishra, Reduction of oxide minerals by hydrogen plasma: An overview *Plasma Chem. Plasma Process.* **2014**, *34*, 1–23.

[11] A. Moezzi, A. M. McDonagh, M. B. Cortie, Zinc oxide particles: Synthesis, properties and applications *Chem. Eng. J.* **2012**, *185*–*186*, 1–22.

[12] J. H. Kim, A. Mirzaei, H. Woo Kim, S. S. Kim, Combination of Pd loading and electron beam irradiation for superior hydrogen sensing of electrospun ZnO nanofibers *Sensors Actuators, B Chem.* **2019**, *284*, 628–637.

[13] D. S. Su, Electron beam induced changes in transition metal oxides *Anal. Bioanal. Chem.* **2002**, *374*, 732–735.

[14] J. Frenkel, Über die Wärmebewegung in festen und flüssigen Körpern *Zeitschrift für Phys.* **1926**, *35*, 652–669.

[15] D. T. Phan, G. S. Chung, Effects of defects in Ga-doped ZnO nanorods formed by a hydrothermal method on CO sensing properties *Sensors Actuators, B Chem.* **2013**, *187*, 191–197.

[16] P. Sundara Venkatesh, P. Dharmaraj, V. Purushothaman, V. Ramakrishnan, K. Jeganathan, Point defects assisted NH3 gas sensing properties in ZnO nanostructures *Sensors Actuators, B Chem.* **2015**, *212*, 10–17.

[17] L. Liao, H. B. Lu, J. C. Li, H. He, D. F. Wang, D. J. Fu, C. Liu, W. F. Zhang, Size dependence of gas sensitivity of ZnO nanorods *J. Phys. Chem. C* **2007**, *111*, 1900–1903.

[18] S. Hettler, E. Kano, M. Dries, D. Gerthsen, L. Pfaffmann, M. Bruns, M. Beleggia, M. Malac, Charging of carbon thin films in scanning and phase-plate transmission electron microscopy *Ultramicroscopy* **2018**, *184*, 252–266.

[19] L. Wang, D. Liu, F. Zhang, Z. Zhang, J. Cui, Z. Jia, Z. Yu, Y. Lv, W. Liu, Dynamics of the charging-induced imaging instability in transmission electron microscopy *Nanoscale Adv.* **2021**, *3*, 3035–3040.

[20] A. Kryshtal, M. Mielczarek, J. Pawlak, Effect of electron beam irradiation on the temperature of single AuGe nanoparticles in a TEM *Ultramicroscopy* **2022**, *233*, 113459.

[21] C. Nuñez Valencia, W. B. Lomholdt, M. H. Leth Larsen, T. W. Hansen, J. Schiøtz, Beam induced heating in electron microscopy modeled with machine learning interatomic potentials *Nanoscale* **2024**, *16*, 5750–5759.

[22] J. Park, K. Bae, T. R. Kim, C. Perez, A. Sood, M. Asheghi, K. E. Goodson, W. Park, Direct Quantification of Heat Generation Due to Inelastic Scattering of Electrons Using a Nanocalorimeter *Adv. Sci.* **2021**, *8*, 1–6.

[23] S. Penner, B. Jenewein, H. Gabasch, B. Klötzer, D. Wang, A. Knop-Gericke, R. Schlögl, K. Hayek, Growth and structural stability of well-ordered PdZn alloy nanoparticles *J. Catal.* **2006**, *241*, 14–19.

[24] I. Langmuir, The mechanism of the catalytic action of platinum in the reactions 2Co + O2= 2Co2 and 2H2+ O2= 2H2O *Trans. Faraday Soc.* **1922**, *17*, 621.

[25] R. Schlögl, Heterogene Katalysatoren – fundamental betrachtet *Angew. Chemie* **2015**, *127*, 3531–3589.

[26] V. Platonov, A. Nasriddinov, M. Rumyantseva, Electrospun ZnO/Pd Nanofibers as Extremely Sensitive Material for Hydrogen Detection in Oxygen Free Gas Phase *Polymers (Basel).* **2022**, *14*, DOI 10.3390/polym14173481.

[27] H. Gabasch, A. Knop-Gericke, R. Schlögl, S. Penner, B. Jenewein, K. Hayek, B. Klötzer, Zn adsorption on Pd(111): ZnO and PdZn alloy formation *J. Phys. Chem. B* **2006**, *110*, 11391–11398.
